# Revision of *Meiodorvillea* Jumars, 1974 (Annelida: Dorvilleidae) including descriptions of three new species from the Southwestern Atlantic Ocean

**DOI:** 10.1371/journal.pone.0264081

**Published:** 2022-03-02

**Authors:** Rafael de Oliveira Bonaldo, Tatiana Menchini Steiner, Antônia Cecília Zacagnini Amaral

**Affiliations:** 1 Programa de Pós-Graduação em Biologia Animal, Instituto de Biologia, Universidade Estadual de Campinas, Campinas, São Paulo, Brazil; 2 Laboratório de Biodiversidade Bêntica Marinha, Departamento de Biologia Animal, Instituto de Biologia, Universidade Estadual de Campinas, Campinas, São Paulo, Brazil; CIIMAR Interdisciplinary Centre of Marine and Environmental Research of the University of Porto, PORTUGAL

## Abstract

*Meiodorvillea* Jumars, 1974 is a little-known genus of Dorvilleidae Chamberlin, 1919, characterized by its small size and reduced appendages and jaw apparatus. A revision of the genus is presented, including analysis of the type material of *M*. *minuta* (Hartman, 1965) and *M*. *apalpata* Jumars, 1974, as well as specimens collected from shelf and slope continental areas in Brazil. A neotype was designated for *M*. *minuta* and its distribution was extended to Brazil. The identity of *M*. *chilensis* (Hartmann-Schröder, 1965) is questioned and three new species from 21 to 1,300.7 meters depth are also described. *Meiodorvillea penhae* **sp. nov.** has furcate chaeta only in the first anterior chaetigers. In contrast, *Meiodorvillea hartmanae* **sp. nov.** has very small palps and asymmetrical thin furcate chaeta and *Meiodorvillea jumarsi* **sp. nov.** has dorsal cirri and geniculate chaeta only in the first anterior chaetigers.

## Introduction

The family Dorvilleidae Chamberlin, 1919 [1] currently comprises 32 genera and about 200 species [2]. Among them, 75 belong to *Ophryotrocha* Claparède & Mecznikow, 1869 [3], of which 35 have been described in the last twenty years [4]. Dorvilleidae includes some of the smallest annelids and shows great morphological heterogeneity, being the only extant family of Eunicida with ctenognath-type jaw apparatus, which consists of two or four rows of symmetrical or subsymmetrical denticulate maxillary plates, upper comb-like jaws, and an unpaired posterior carrier-like structure [2].

Taxonomical and ecological features of some genera are well-known, such as *Ophryotrocha* [5, 6]. On the other hand, there is an overall paucity of information on Dorvilleidae, with 13 of the genera being monospecific likely due to their rarity in intertidal to abyssal environments. Despite the recent increase in knowledge on the diversity of dorvilleids, the family interrelationships remain unclear, mainly due to the low level of morphological details in the description of the known species, combined with the lack of specialized taxonomists and studies on its phylogeny.

Free-living dorvilleids range from small interstitial to large species living in unconsolidated bottoms, including sand, mud, and hard substrates, while some are commensals or parasites [2]. Ten species have been recorded in Brazil: *Dorvillea moniloceras* (Moore, 1909) [7], *D*. *sociabilis* (Webster, 1879) [8], *Ophryotrocha puerillis* Claparède & Mecznikow, 1869 [3], *Pettiboneia sanmatiensis* Orensanz, 1973 [9], *Protodorvillea biarticulata* Day, 1963 [10], *P*. *kerfersteini* (McIntosh, 1869) [11], *Schistomeringos annulata* (Moore, 1906) [12], *S*. *anoculatus* (Hartman, 1965) [13], *S*. *longicornis* (Ehlers, 1901) [14], and *S*. *rudolphi* (delle Chiaje, 1828) [15, 16]. The presence of *Meiodorvillea* has not been reported in Brazilian coast to date.

*Meiodorvillea* is composed of very small and simple-bodied species reaching few centimeters in length and few morphological structures for identification. The genus was erected by Jumars [17] to include *M*. *minuta* (Hartman, 1965) [13], *M*. *chilensis* (Hartmann-Schröder, 1965) [18], and *M*. *apalpata* Jumars, 1974 [17]. *Meiodorvillea* differs from *Protodorvillea* in the extreme reduction of palps and maxillary apparatus with two rows of maxillary plates or “denticles”. *Meiodorvillea minuta* was described from New England (USA) and *M*. *apalpata* from San Diego (USA). *Meiodorvillea chilensis* is known only from the original description, based on a single specimen from Golfo de Ancud, Chile. Two unnamed species are described by Wolf [19], from Gulf of Mexico (Florida and Texas, USA). *Meiodorvillea* is a little-known genus with studies restricted to original taxonomic descriptions of species, in addition to Jumars [17], when the genus was erected. It occurs from 21 to 1300 meters depth, lacking adequately fixed samples for molecular studies, and rare in scientific collections, so that its diversity is currently underestimated. In this scenario, relevant studies on the taxonomy, morphology, ecology, biology, ontogeny, and behavior, among others, are absent, leading most specimens from museums to be identified either as *Meiodorvillea* sp. or as *M*. *minuta*.

This study intends to review morphologically the genus *Meiodorvillea* by designating a neotype for *M*. *minuta* and by describing three new species, extending the occurrence of the genus to Brazil. Some morphological characters used in the taxonomy of the genus, as well as its worldwide distribution, are also discussed.

## Materials and methods

### Sampling

Specimens were collected along the Southwestern Atlantic Ocean during two major Brazilian oceanographic research projects carried out between 2008 and 2012, in soft-bottoms from 12 to 3.301 m depth: the Assessment of the Environmental Heterogeneity of the Campos Basin (HABITATS) [20] and the Environmental Characterization of the Espírito Santo Basin (AMBES). The collected specimens were fixed in 4% formalin and preserved in 70% ethanol.

### Morphological analysis

The whole body and parapodia from anterior, median and posterior regions of specimens were analyzed under both Zeiss SteREO Discovery V.2.0 and Zeiss Axioskop 2 Plus optical microscopes, and scanning electron microscope (SEM), model JEOL JSM-5800 LV at the Laboratório de Microscopia Eletrônica, Instituto de Biologia, Universidade Estadual de Campinas (UNICAMP). Specimens for SEM were immersed 15 minutes in increasing concentrations of ethanol (80, 90, and 95%), followed by 15, 30, and 60 minutes in absolute ethanol. Critical point drying (Balzers CPD-30) was carried out at 37°C and 70 bars, followed by gold coating (Sputter Coater SPD-050) [21].

Jaw apparatuses were prepared for optical microscopy using three methods. 1. Cutting anterior end with about four chaetigers, whitening with 10% KOH (potassium hydroxide) for one to two hours, depending on the specimen size, and mounting on slides with glycerin. 2. Cutting anterior end with about four chaetigers and dissolving the soft tissues with 30% NaClO (sodium hypochlorite), then washing until degradation, and mounting on slides with Hoyer (trichloroacetaldehyde) or Aquatex®. 3. Drying entire specimens on slides and observing by transparency. Jaw apparatuses of the three new species were analyzed and described on non-type specimens, to avoid harming holotypes’ integrity. Drawings were made with camera lucida and photos with ZEISS AxioCam MRc, both attached to optical microscope.

Type and some non-type specimens of *M*. *minuta* and *M*. *apalpata* from the Polychaete Collection (LACM-AHF Poly) of the Natural History Museum of Los Angeles County (NHMLAC) and the Polychaeta Collection (USNM) of the National Museum of Natural History of Smithsonian Institution were not dissected, neither used for SEM, to avoid damaging. The specimens of the new species and *M*. *minuta* from Brazil are deposited at the Polychaeta Collection (ZUEC-POL) of the Museu de Diversidade Biológica (MDBio), Instituto de Biologia, Universidade Estadual de Campinas (IB/UNICAMP), São Paulo, Brazil.

### Nomenclatural acts

The electronic edition of this article conforms to the requirements of the amended International Code of Zoological Nomenclature, and hence the new names contained herein are available under that Code from the electronic edition of this article. This published work and the nomenclatural acts it contains have been registered in ZooBank, the online registration system for the ICZN. The ZooBank LSIDs (Life Science Identifiers) can be resolved and the associated information viewed through any standard web browser by appending the LSID to the prefix “http://zoobank.org/”. The LSID for this publication is: urn:lsid:zoobank.org:pub:D969E420-552D-41DC-9FEB-A5131142E9F7. The electronic edition of this work was published in a journal with an ISSN, and has been archived and is available from the following digital repositories: PubMed Central, LOCKSS and Repositório da Produção Científica e Intelectual da UNICAMP.

## Results

### Systematics

Family **Dorvilleidae** Chamberlin, 1919

Genus ***Meiodorvillea*** Jumars, 1974

#### Type-species

*Protodorvillea minuta* Hartman, 1965 [13]

#### Type locality

Block Canyon, off New England (USA), 39°58’24"N, 70°40’18"W

#### Amended diagnosis

Prostomium pear-shaped. One pair of simple and clavate dorsal antennae (Fig 3A and 3B). One pair of simple and clavate lateral palps, sometimes absent, shorter than antennae (Fig 19A and 19B). Jaw apparatus (Fig 12) with paired and medially fused butterfly-shaped mandibles. Maxillae with one pair of V-shaped dorsal carrier-like structures and one pair of ventral basal plates, each one fused posteriorly, and free from each other. Inner margin of basal plates smooth, rarely dentate. Ten to 15 pairs of maxillary plates in two rows, anteriorly to the basal plates, denticulate on its margin, absent in *M*. *apalpata*. Two peristomial rings. Small dorsal cirri sometimes present, without acicula (Fig 4A and 4B). Small ventral cirri present (Fig 10). Supra-acicular chaetae: (1) capillaries (Fig 11A), (2) geniculates (Figs 11C and 21A) and (3) furcates (Figs 11B, 11H and 16A-16D). Sub-acicular chaetae: (4) compound falcigers and/or spinigers (Figs 11E–11G and 21C) and (5) cultriforms (Figs 16H and 21D). Pygidium with two pairs of cirri (Fig 9E and 9F).

#### Remarks

Jumars [17] erected *Meiodorvillea* naming *M*. *minuta* as type species, describing *M*. *apalpata*, and raising *M*. *chilensis* (previously *Protodorvillea gaspeensis chilensis* Hartmann-Schröder, 1965 [18]) to species. *Meiodorvillea chilensis* and *P*. *gaspeensis* Pettibone, 1961 [22] have short biarticulated palps, so that the latter was considered to belong to a new genus [23] and assigned as type species of *Marycarmenia* Núñez, 1998 [24]. The jaw apparatus of this genus is markedly different from *Meiodorvillea* in the absence of maxillary carrier-like structures and the presence of denticles on the anterior margin of mandibles [24]. *Meiodorvillea chilensis* was described based on one specimen, and although it was well described and illustrated, the jaw apparatus was not examined, which makes its determination questionable [19]. Given the lack of information about its jaw apparatus, *M*. *chilensis* must remain as *species inquirenda* possibly also belonging to *Marycarmenia*. The character “biarticulated palps”, proposed by Jumars [17], has been removed from the diagnosis, as it only occurred in *M*. *chilensis*.

Eibye-Jacobsen & Kristensen [23] proposed the erection of a new genus to *M*. *apalpata*, suggesting that it could be a sister group of *Meiodorvillea sensu stricto*. However, Jumars [17] stated that no ‘free denticles’ (maxillary plates) remained in the specimens, which did not allow distinguishing a real absence from a handling artifact. Therefore, maxillary plates could be present, but this could not be assessed in handled specimens. Although *M*. *apalpata* is the only one with lacking palps, it shares characters, such as absence of dorsal cirri, shape of mandibles, absence of denticles on its anterior margin, and presence of maxillary carrier-like structures. Furthermore, the absence of furcate chaeta is also verified in other species, either in the anterior or posterior region. As proposed by Wolf [19], our analysis found no significant morphological differences to support the erection of the new genus proposed by Eibye-Jacobsen & Kristensen [23]. Therefore, *M*. *apalpata* should remain within *Meiodorvillea* until more specimens will be carefully examined, especially regarding the jaw apparatus.

The present revision allowed including new morphological character states in the generic diagnosis, such as presence and shape of geniculate, furcate and cultriform chaetae and details of jaw apparatus.

**Fig 1:**
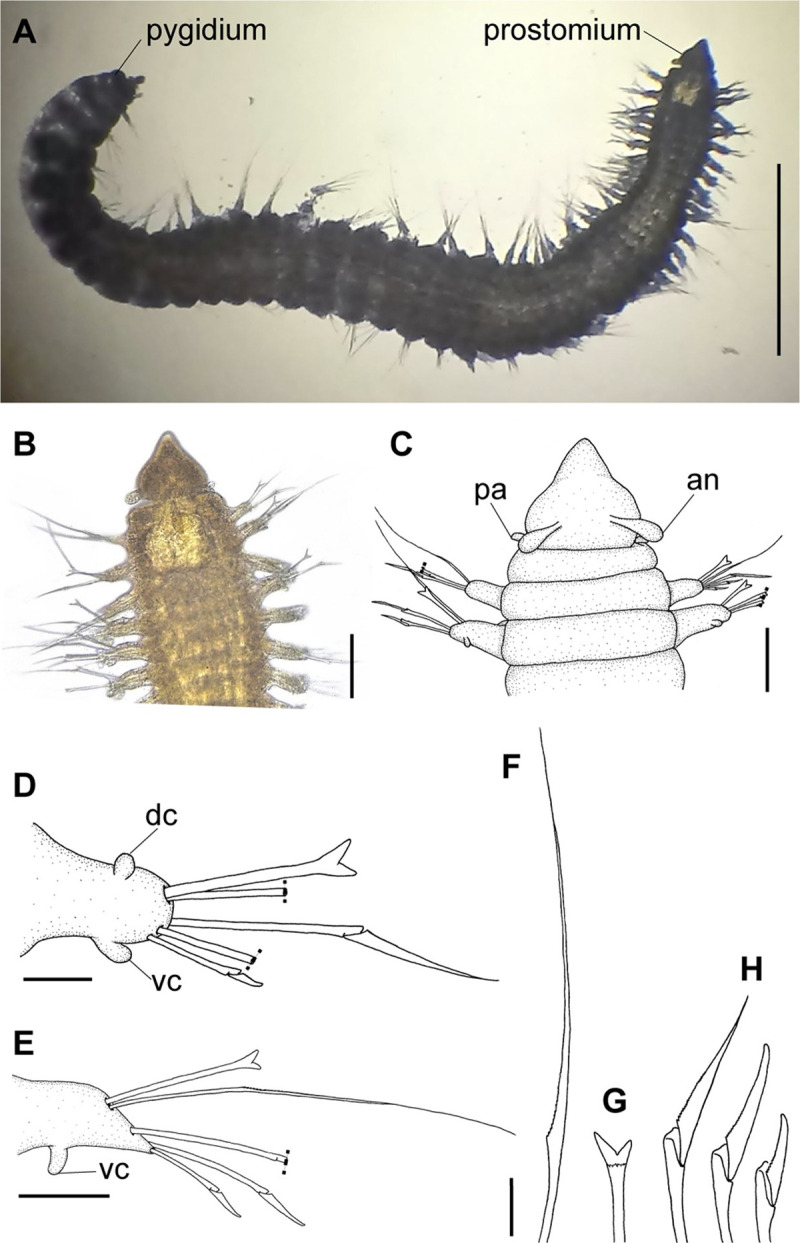
*Meiodorvillea minuta* (Hartman, 1965), neotype (LACM-AHF Poly 12561). (A) complete specimen, dorsal view; (B,C) anterior region, dorsal view; (D) parapodium 4, anterior view; (E) parapodium 12, anterior view; (F) capillary chaeta; (G) furcate chaeta; (H) compound chaetae. Dash lines represent broken chaeta. Abbreviations: an = antenna; dc = dorsal cirrus; pa = palp; vc = ventral cirrus. Scale bars: (A) = 0.5 mm; (B) = 80 μm; (C) = 50 μm; (D) = 20 μm; (E) = 40 μm; (F,G,H) = 12.5 μm.

The posterior peristomial ring frequently covers the anterior ring in specimens where the contraction of the anterior region is markedly visible (Fig 14A and 14B), which apparently demonstrates the presence of one ring (Figs 1C and 3B) [13, 17]. Although this is not a diagnostic character, this variation is mentioned in the descriptions as being rather variable and dependent on the state of contraction caused by fixation of the specimens.

The free denticles, terminology adopted by Jumars [17], Oug [25], and Eibye-Jacobsen & Kristensen [23], are here referred as the paired maxillary plates (placed anteriorly to basal plates), following Wolf [26] and Purschke [27]. Each maxillary plate contains denticles on its margin.

### *Meiodorvillea minuta* (Hartman, 1965)

(Figs 1–6, Table 1)

**Fig 2:**
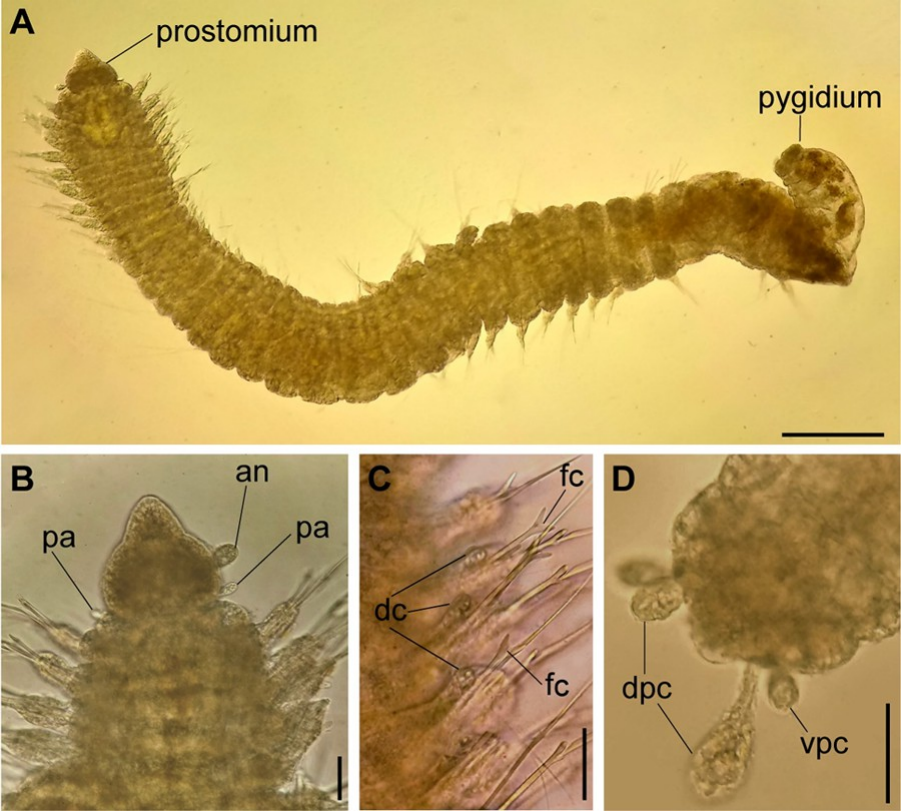
*Meiodorvillea minuta* (Hartman, 1965), ZUEC-POL. (A) complete specimen, dorsal view; (B) anterior region, dorsal view; (C) parapodia from anterior region, dorsal view; (D) pygidium, ventral view. Abbreviations: an = antenna; dc = dorsal cirrus; dpc = dorsal pygidial cirrus; fc = furcate chaeta; pa = palp; vpc = ventral pygidial cirrus. Scales: (A) = 200 μm; (B,C) = 62.5 μm; (D) = 31.25 μm.

**Fig 3:**
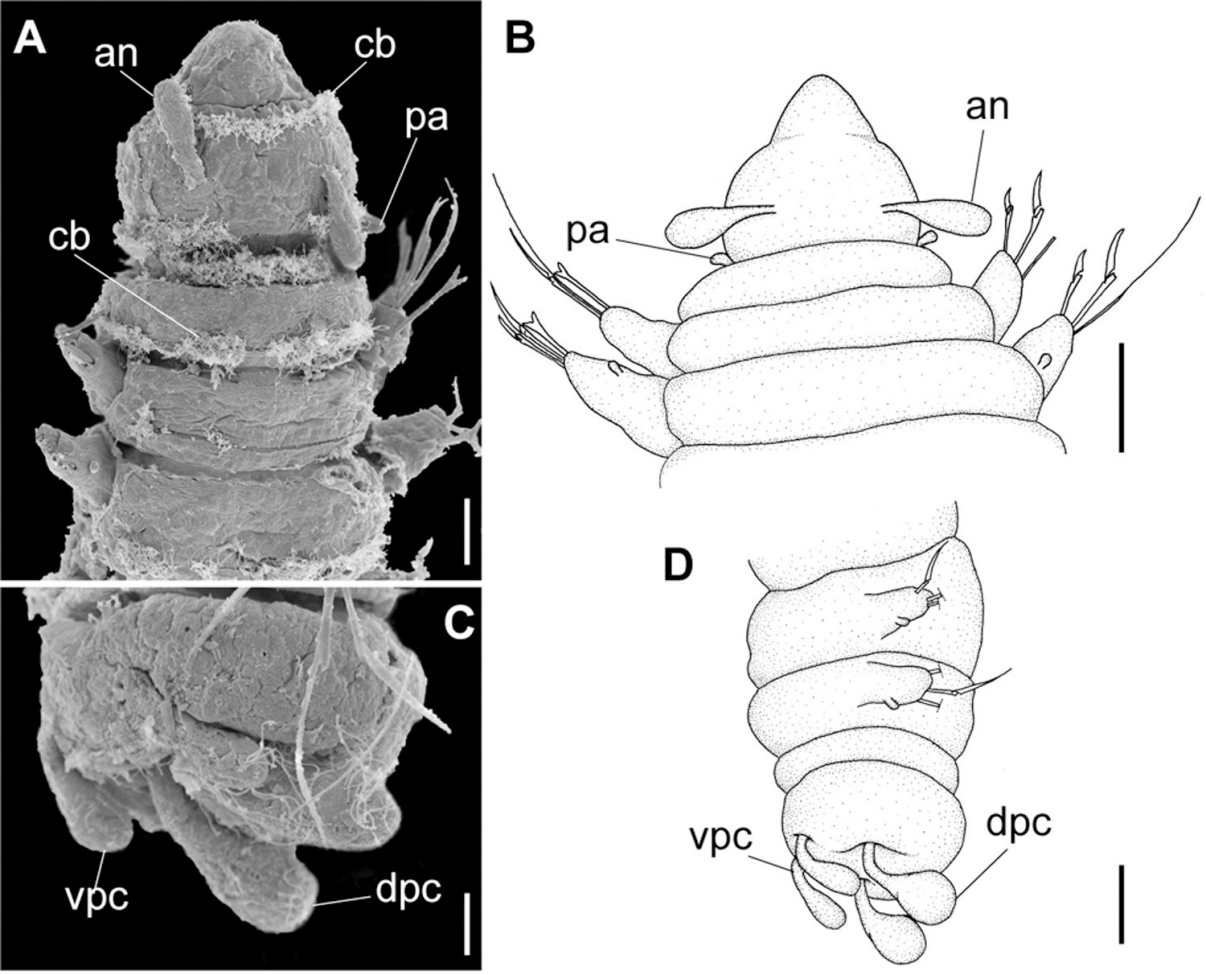
*Meiodorvillea minuta* (Hartman, 1965), ZUEL-POL. (A,B) anterior region, dorsal view; (C,D) pygidium, lateral view. Abbreviations: an = antenna; dpc = dorsal pygidial cirrus; pa = palp; vpc = ventral pygidial cirrus. Scales: (A) = 20 μm; (B) = 25 μm; (C) = 10 μm; (D) = 15.6 μm.

**Fig 4:**
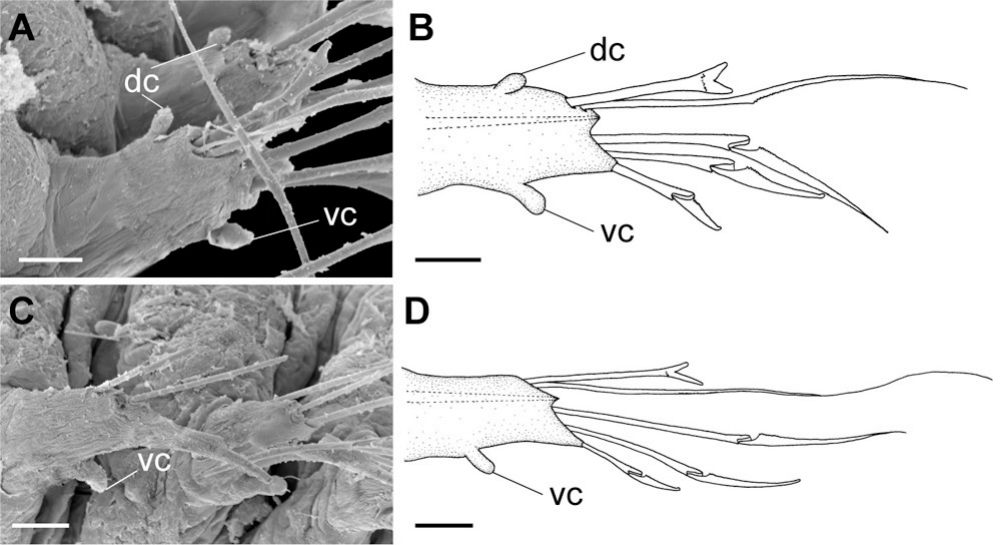
*Meiodorvillea minuta* (Hartman, 1965), ZUEC-POL. (A,B) parapodia from anterior region; (C,D) parapodia from posterior region; all anterior view. Abbreviations: dc = dorsal cirrus; vc = ventral cirrus. Scales: (A,B) = 10 μm; (C,D) = 15.6 μm.

**Fig 5:**
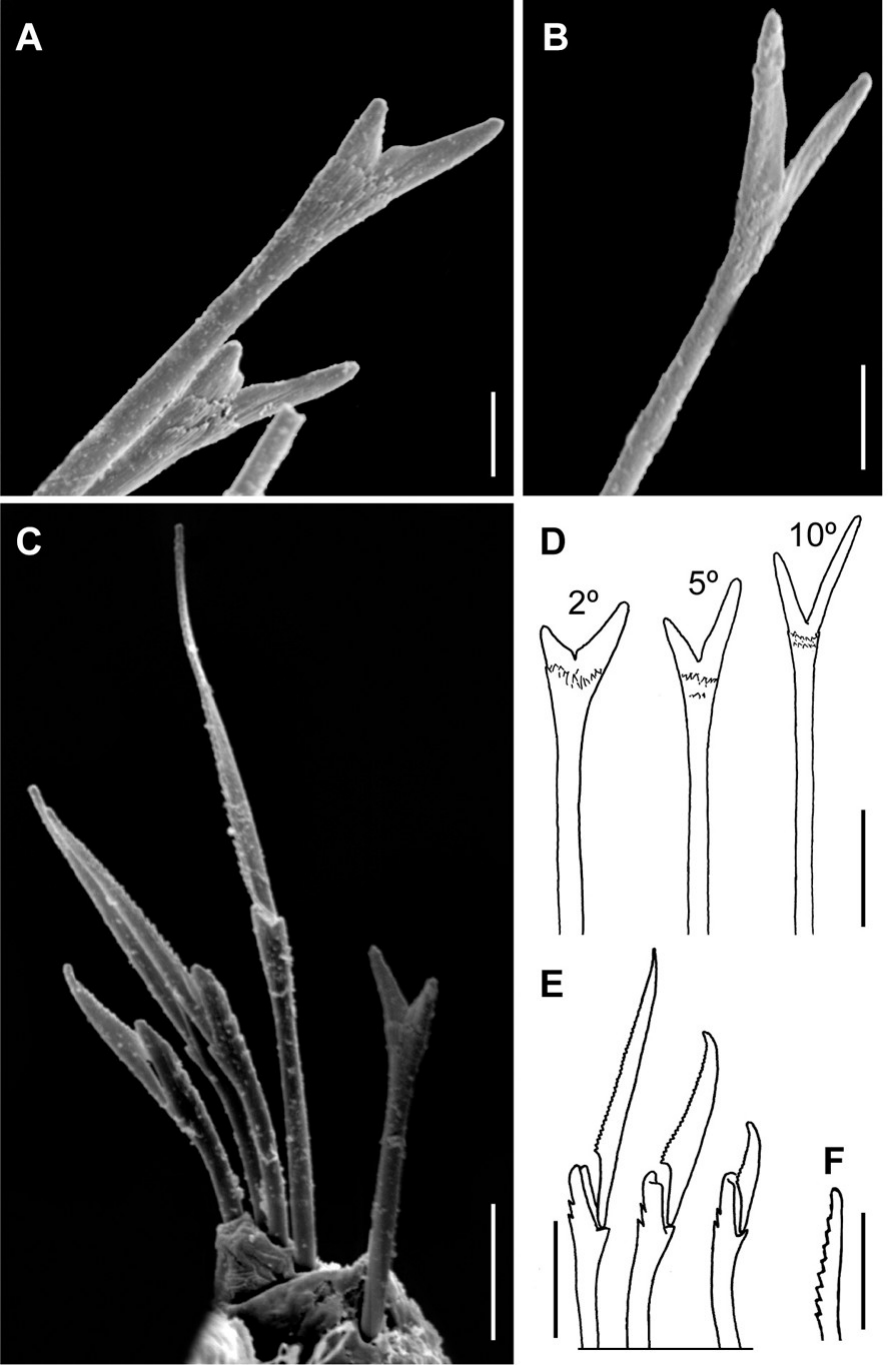
*Meiodorvillea minuta* (Hartman, 1965), ZUEC-POL. (A) furcate chaeta from anterior region; (B) furcate chaeta from posterior region; (C,E) compound chaetae; (D) morphology of furcate chaeta, from chaetigers 2, 5, and 10; (F) cultriform chaeta. Scales: (A,B) = 5 μm; (C) = 10 μm; (D,E,F) = 6.25 μm.

**Fig 6:**
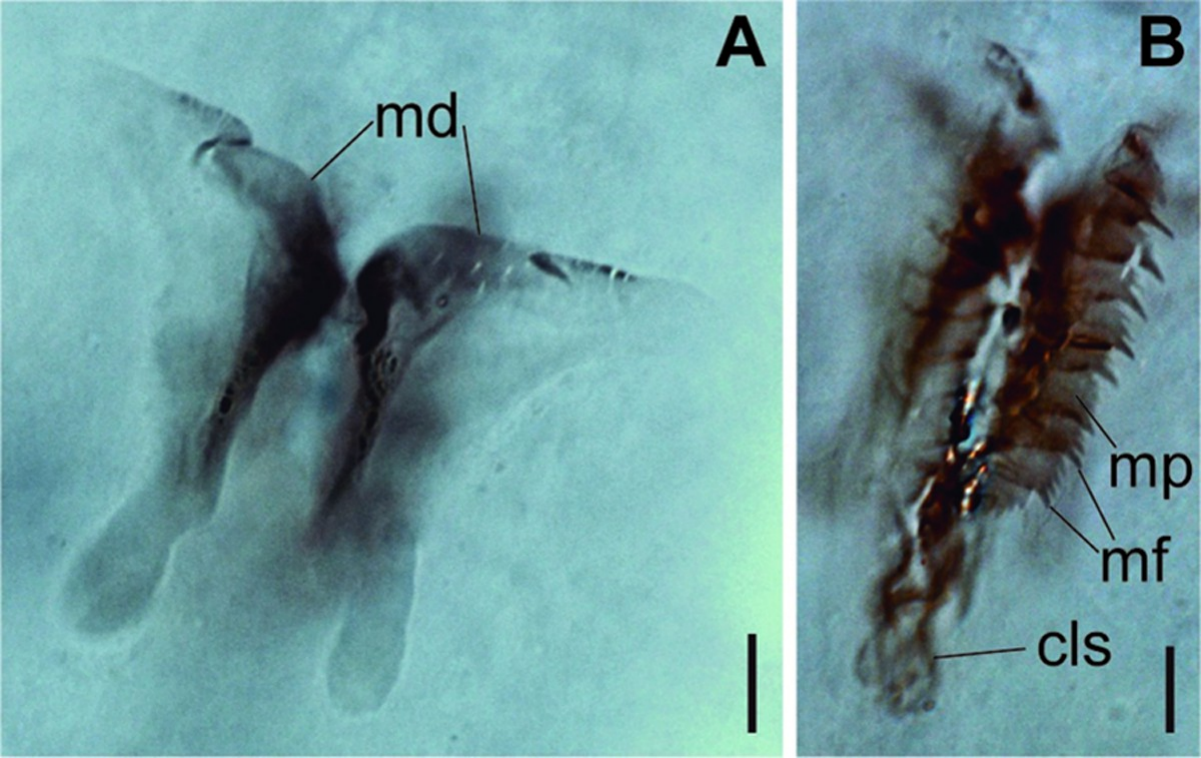
*Meiodorvillea minuta* (Hartman, 1965), ZUEC-POL. (A) mandibles, dorsal view; (B) maxillae, dorsal view. Abbreviations: md = mandibles; mp = maxillary plates; mf = main fang; cls = carrier-like structure. Scales: (A,B) = 10 μm.

**Table 1:** Main morphological differences and distribution of *Meiodorvillea* species.

SPECIES	PALPS	PRESENCE OF DORSAL CIRRUS	FURCATE CHAETAE			GENICULATE CHAETAE	Y-SHAPED SHAFT OF DORSAL COMPOUND	DISTRIBUTION
			PRESENCE/ABSENCE	SYMMETRY OF PRONGS				
*Meiodorvilleaminuta*	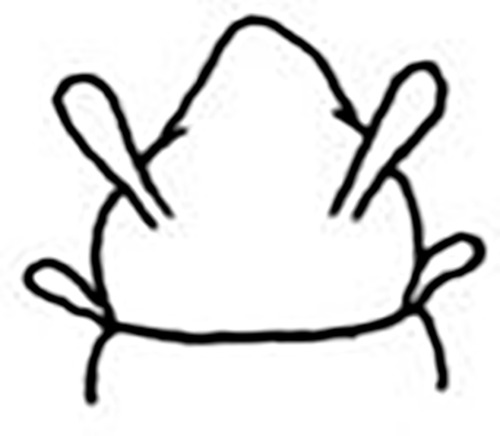 small clavate	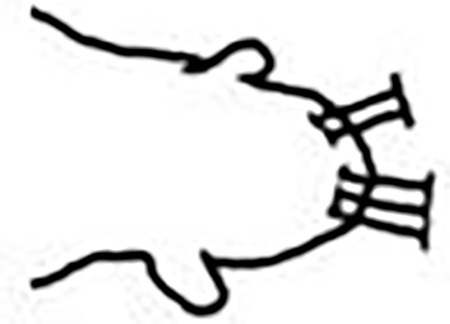 chaetigers 2 to 5–9, smaller than ventral	present on all chaetigers	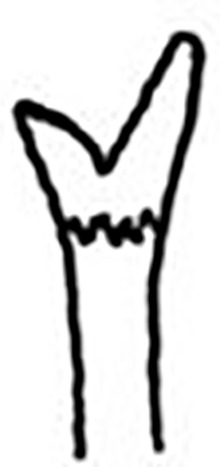 asymmetrical		absent	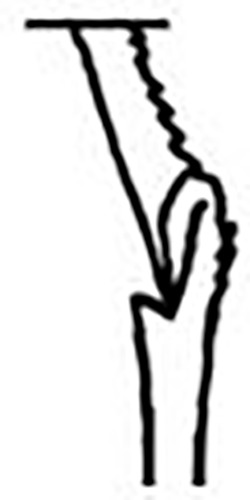 absent	New England and North Carolina, USA; Espírito Santo and Rio de Janeiro States, Brazil (97–1,050 m)
*Meiodorvilleaapalpata*	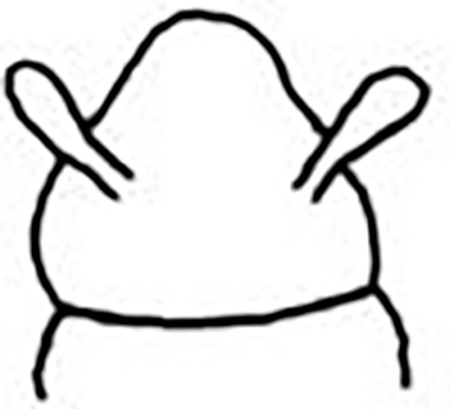 absent	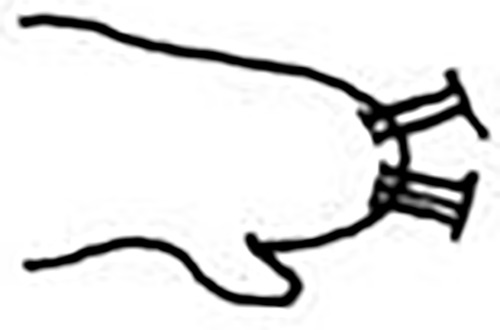 absent	absent			present on all chaetigers	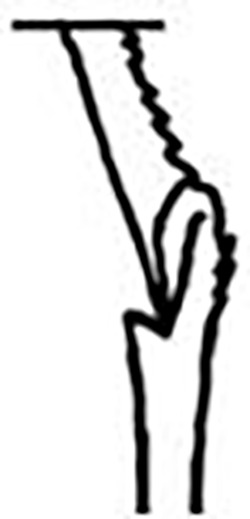 absent	east coast of California, San Diego Trough (1,223–1,224 m)
*Meiodorvilleapenhae* **sp. nov.**	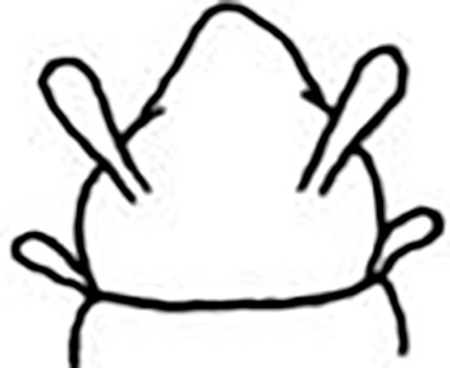 small clavate	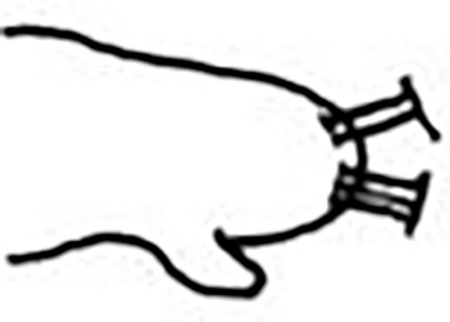 absent	present on chaetigers 1 to 7–9		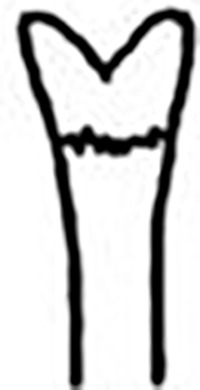 symmetrical	replace furcate after chaetiger 7–9	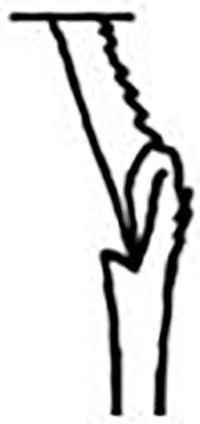 absent	Espírito Santo and Rio de Janeiro States, Brazil (21–158 m)
*MeiodorvilleaHartmanae* **sp. nov.**	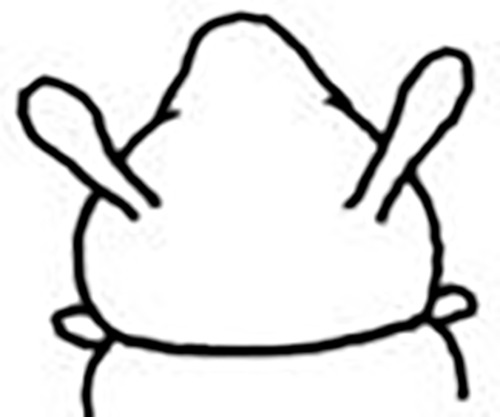 very small clavate	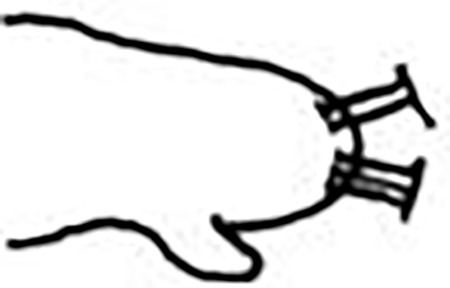 absent	present on all chaetigers		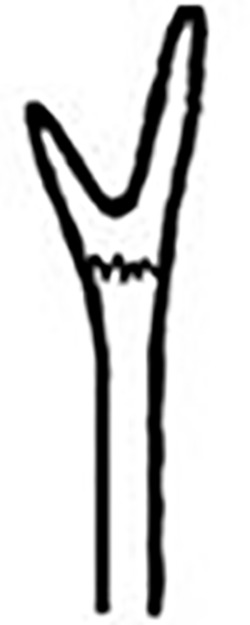 asymmetrical	absent	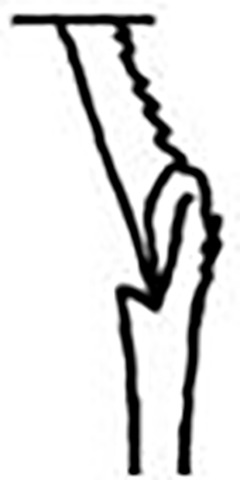 absent	Espírito Santo and Rio de Janeiro States, Brazil (964,8–1,300.7 m)
*Meiodorvilleajumarsi* **sp. nov.**	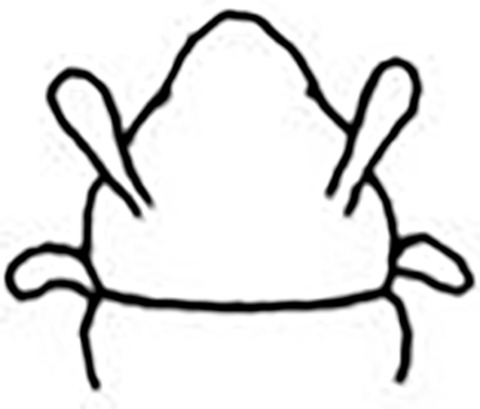 small clavate	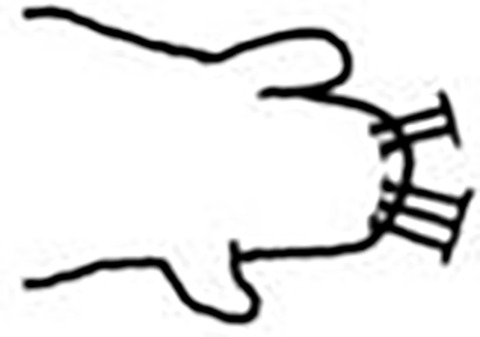 chaetigers 2 to 5–9, larger than ventral	replace geniculate after chaetigers 7–13		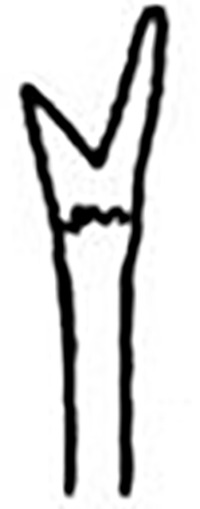 asymmetrical	present on chaetigers 1 to 7–13	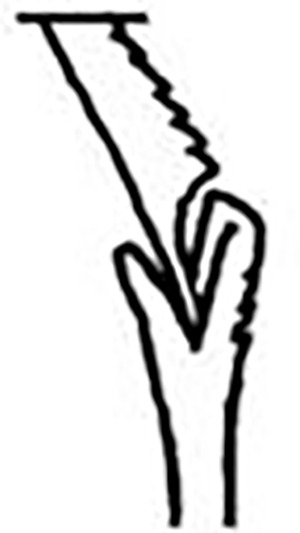 present	Espírito Santo and Rio de Janeiro States, Brazil (41–432 m)

***Protodorvillea minuta*** Hartman, 1965 [13]: 125–127, plate 23

***Meiodorvillea minuta***–Jumars, 1974 [17]: 119–120, Fig 9

#### Type locality

Block Canyon, New England (USA), upper continental slope, 39°58’24"N, 70°40’18"W, 300 m depth.

#### Type material examined

Neotype: LACM-AHF Poly 12561 39°58’24"N 70°40’18"W, 300 m, very fine sand, 28 Aug 1962. Paratypes: LACM-AHF Poly 692 (6 specimens) 39°58’24"N 70°40’18"W, 300 m, very fine sand, 28 Aug 1962. (m = meters depth).

#### Other material examined

State of North Carolina–USNM 1008896 (8 specimens) 34°15’2.16"N 75°43’40.08"W, 1,019 m, mud, 27 Mar 1984. State of Espírito Santo–ZUEC-POL 21403 (1 spec) 21°11’12.228"S 40°12’51.745"W, 683 m, 04 Feb 2009; ZUEC-POL 21404 (1 spec) 20°36’1.61"S 39°51’39.15"W, 1,003 m, mud, 18 Jun 2013; ZUEC-POL 21392 (1 spec) 21°11’3.022"S 40°12’19.168"W, 790.2 m, 05 Jul 2008. State of Rio de Janeiro–ZUEC-POL 21393 (1 spec) 21°47’26.324"S 40°2’13.825"W, 730.5 m, 28 Jun 2008; ZUEC-POL 21394 (1 spec) 22°36’25.559"S 40°22’28.988"W, 698.1 m, 29 Jan 2009; ZUEC-POL 21395 (1 spec) 22°36’27.781"S 40°22’30.727"W, 695.4 m, 29 Jan 2009; ZUEC-POL 21396 (1 spec) 21°41’11.649"S 40°2’20.690"W, 699.4 m, 07 Jul 2008; ZUEC-POL 21397 (1 spec) 22°19’2.381"S 40°5’27.062"W, 383.8 m, 30 Jan 2009; ZUEC-POL 21398 (1 spec) 22°33’35.143"S 40°26’37.449"W, 401 m, 31 Jan 2009; ZUEC-POL 21399 (1 spec) 23°39’21.880"S 41°18’33.045"W, 692.7 m, 28 Jan 2009; ZUEC-POL 21400 (1 spec) 23°37’58.489"S 41°19’41.504"W, 400.5 m, 01 Feb 2009; ZUEC-POL 21401 (4 spec) 21°56’11.912"S 39°57’45.190"W, 705.2 m, 28 May 2008; ZUEC-POL 21402 (3 spec) 22°19’10.211"S 40°5’42.884"W, 400 m, 10 Feb 2009. (m = meters depth).

#### SEM material

State of Espírito Santo–ZUEC-POL 21405 (2 spec): 19°37’45.14"S 39°3’58.75"W, 1,050 m, mud, 25 Jun 2013 and 20°14’17.95"S 39°48’34.35"W, 395 m, mud, 19 Jun 2013. (m = meters depth)

#### Diagnosis

One pair of antennae and one pair of palps. Dorsal cirrus papilliform from chaetigers 2 to 5–9. Ventral cirrus papilliform, absent in the first chaetiger. Chaetae capillary, furcate asymmetrical, dorsalmost compound spiniger or falciger, median and ventralmost falcigers, and cultriform occasionally in last chaetigers in some specimens, replacing the ventralmost compound. Two pairs of pygidial cirri.

#### Designation of neotype

Hartman [13] listed the material examined of *M*. *minuta* as ‘Records: C 1 (3); S1 3 (11, TYPE); S1 4 (6); D 1 (4)’, mentioning off New England as the type locality, but not designating holotype and paratypes. Jumars [17] mentioned and re-examined the holotype, without clarifying the exact type locality.

The catalog of the Polychaete Collection of the Natural History Museum of Los Angeles County (NHMLAC) has registered the holotype, examined by Jumars [17], in the lot LACM-AHF Poly 0691 and seven paratypes in LACM-AHF Poly 0692. Both lots were assigned later the Hartman’s [13] publication, the holotype by assistants of Olga Hartman and paratypes by Kristian Fauchald (Leslie Harris, NHMLAC, pers com.), at the time a graduate student, but who has become one of the greatest 20th century polychaetologists. Although Jumars [17] examined and described the holotype, the vial was empty, so that the holotype is missing.

Both R/V Atlantis Expedition Gay Head (Bermuda Transect)’s data [13] and the NHMLAC’s catalog show that the type locality of *M*. *minuta* is the Station Sl 3 (letter ‘l’, instead number ‘1’), meaning Station Slope 3 (east of upper end of Block Canyon, in New England upper continental slope), at 39°58’24"N, 70°40’18"W, 300 meters deep, collected at 28 August 1962. Thus, in accordance with ICZN’s Articles 75.1 and 75.3.1 [28], we designate the neotype LACM-AHF Poly 12561, selected from the paratypes of LACM-AHF Poly 0692, clarifying the type locality of *M*. *minuta*.

In the same way, considering Articles 75.3.2 and 75.3.3 of the ICZN [28], the neotype is named to ensure morphological data sufficient for recognition of the species, clarifying the correct presence and distribution of dorsal and ventral cirri, not clearly described by Hartman [13] and Jumars [17]. Likewise, *M*. *minuta* represents the species type of the genus, requiring the correct registration of its morphological characters.

#### Redescription of neotype

Complete specimen, 43 chaetigers, 2.5 mm long, median region 0.25 mm wide, excluding parapodia; body width almost uniform, anterior and posterior regions slightly narrower (Fig 1A). Color in ethanol pale yellow.

Prostomium pear-shaped, as long as wide, anterior half depressed, posterior half globular, as long as the first two segments (Fig 1A–1C). Eyes absent. One pair of clavate antennae inserted dorsolaterally on middle posterior half of prostomium, 2/3 of prostomium length. One pair of small and clavate palps inserted laterally at prostomium base, 1/3 as long as antennae (Fig 1B and 1C).

Jaw apparatus not dissected. Mandibles butterfly-shaped medially fused, anterior region enlarged with smooth margins without free or fused teeth, posterior region slender and curved. Basal plates of maxillae with smooth inner margin; two rows of 12–15 pairs of denticulate maxillary plates [17].

Two peristomial rings, posterior wider and longer than anterior, covering it dorsally. Chaetigers wide and short, anterior narrower than median and posterior (Fig 1A).

Cylindrical parapodia, first pair smaller than the following ones, gradually tapering towards posterior region (Fig 1A, 1D and 1E). Small and rounded dorsal cirrus on chaetigers 2 to 9 (Fig 1D), inserted slightly distally on the parapodium, absent thereafter. Small papilliform ventral cirrus in all parapodia, absent in the first one (Fig 1E), inserted medially in the parapodium; ventral cirrus larger than dorsal on same parapodium.

Supra-acicular chaetae: (1) one long and serrated capillary (Fig 1F), with a small limb anteriorly, longer and thinner in median and posterior regions; (2) one thick furcate with short triangular prongs, asymmetrical in size and shape and serrated base (Fig 1G), thinner and longer in median and posterior regions. From chaetiger 9, two capillaries and one furcate. Sub-acicular chaetae: (3) three compound heterogomphs (Fig 1H), distal end of shafts serrated and blades unidentate with serrated cutting edge; dorsalmost longest with falcigerous or spinigerous blade, median falcigerous, ventralmost falcigerous and shortest. Cultriform chaeta absent. Chaetae from median and posterior regions longer and slender. One internal thick acicula.

Pygidium rounded and smaller than previous chaetigers. Two pairs of clavate pygidial cirri (Fig 1A), dorsal pair with 1.5 times as long as pygidium, ventral pair as long as the pygidium.

#### Variation

Complete specimens with 31–52 chaetigers, some of them with posterior moniliform chaetigers. ZUEC-POL complete specimens 1.7–4 mm long, maximum 0.23 mm wide.

Antennae 1/3–2/3 the length of the prostomium. Some specimens with posterior peristomial ring covering the anterior dorsally. Dorsal cirri from chaetigers 2 to 5–9. Two capillary chaetae in median and posterior regions in most specimens and presence of serrated cultriform chaetae in paratypes and Brazilian specimens, replacing the ventralmost compound (Fig 5F).

Anterior region, antennae, palps (Figs 2A, 2B, 3A and 3B), peristomial rings, and organization of parapodia and pygidium (Figs 2D, 3C and 3D) of ZUEC-POL specimens are in accordance with the above description, except for a ciliary band, viewed under SEM, between prostomium halves (Fig 3A), base of peristomial rings and anterior chaetigers (Figs 2B, 3A and 3B). Chaetae and both organization on parapodia and form agree with description (Fig 5).

Jaw apparatus of ZUEC-POL specimens with ventrally and medially fused mandibles, anterior region enlarged with smooth margins without free or fused teeth, posterior region slender and curved (Fig 6A). Basal plates of maxillae with smooth inner margin, two subsymmetrical rows with 10–12 pairs of free rectangular and denticulate maxillary plates, each one with one posterior main fang and usually four anterior teeth; anterior and posteriormost plates larger and rounded, with small and more numerous teeth (Fig 6B).

#### Remarks

Hartman [13] described the parapodial cirri as being obscure and reduced to slight projections in all parapodia, which differs from the analysis performed on the type series and non-type material. The LACM-AHF Poly specimens did not have cirri in parapodium 1, but rather a small papilliform ventral cirrus from the 2, in addition to a small and rounded dorsal cirrus from the parapodia 2 to 7–9. The length of antennae in the original description is not accurate, being actually 2/3 the length of prostomium. The furcate chaetae become slender and longer with asymmetrical prongs in median and posterior regions, character not mentioned by Hartman [13].

The jaw apparatus of the type series was not analyzed, but the morphology of the ZUEC-POL specimens agrees with Jumars’s [17] description.

#### Geographic distribution and bathymetric range

Off New England (USA), 97–508.7 m, very fine sand [13]; continental slope off North Carolina (USA), 850–1,019 m [29, 30]; Southwestern Atlantic Ocean, States of Espírito Santo and Rio de Janeiro (Brazil), 383.8–1,050 m, mud.

### *Meiodorvillea apalpata* Jumars, 1974

(Fig 7, Table 1)

**Fig 7:**
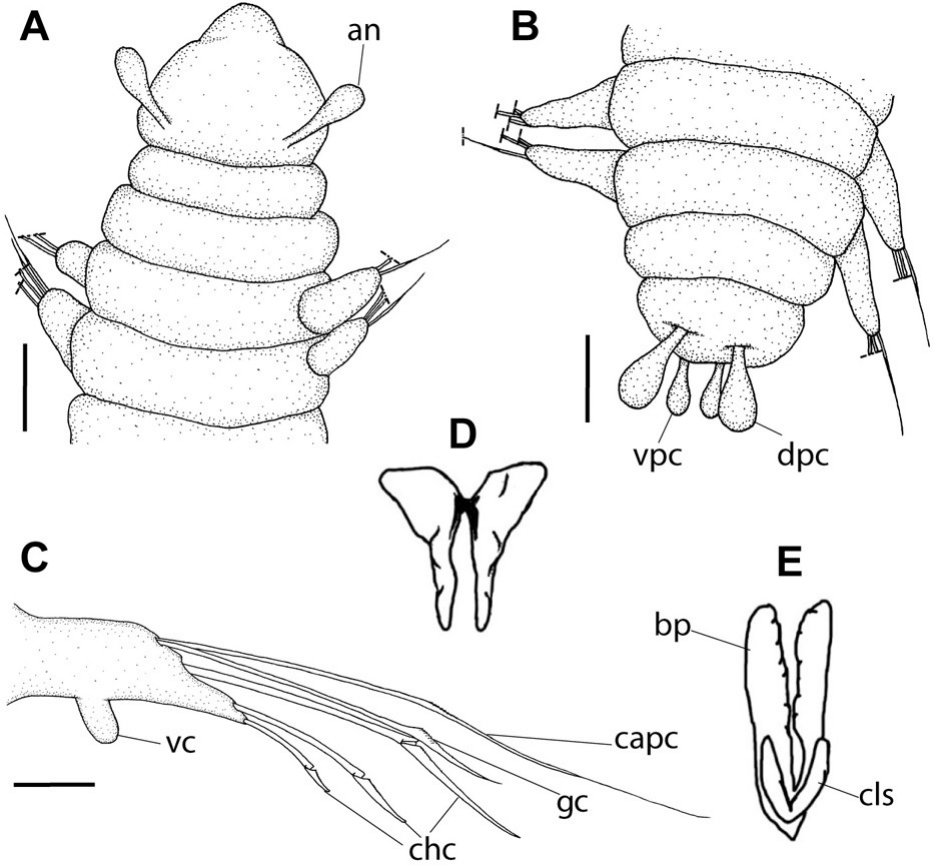
*Meiodorvillea apalpata* Jumars, 1974, LACM-AHF Poly. (A) anterior region, dorsal view; (B) pygidium, dorsal view; (C) parapodium from posterior region, anterior view; (D) mandibles; (E) basal plates and maxillary carrier-like structures. Dash lines represent broken chaeta. Abbreviations: an = antenna; capc = capillary chaeta; chc = compound heterogomph chaeta; dpc = dorsal pygidial cirrus; gc = geniculate chaeta; vc = ventral cirrus; vpc = ventral pygidial cirrus. Scale bars: (A,B) = 50 μm; (C) = 20 μm. (D,E): modified from Jumars [17] (no scale bars in the original figures).

### *Meiodorvillea apalpata* Jumars, 1974 [17]: 121–122, Fig 10

#### Type locality

San Diego Trough region, 32°28.2’N 117°29.8’W, Coronado Sea, California, United States of America, 1,224 m depth.

#### Type material examined

Holotype: LACM-AHF Poly 1086, 32°28.2’N 117°29.8’W, 1,224 m, silty mud, 06 Dec 1972. Paratypes: LACM-AHF Poly 1087 (1 spec) 32°28.2’N 117°29.8’W, 1,224 m, silty mud, 06 Dec 1972; LACM-AHF Poly 1088 (1 spec) 32°28.2’N 117°29.8’W, 1224 m, silty mud, 12 Dec 1969 and LACM-AHF Poly 1089 (1 spec) 32°38.9’N 117°30.1’W, 1,223 m, silty mud, 05 Jan 1972. (m = meters depth).

#### Diagnosis

One pair of antennae. Palps and dorsal cirri absent. Ventral cirrus papilliform, absent in the first chaetiger. Chaetae capillary, geniculate, all three compound falcigers, and cultriform in last chaetigers in some specimens, replacing ventralmost compound. Two pairs of pygidial cirri.

#### Redescription of holotype

Complete specimen in poor condition, 47 chaetigers in three fragments, anterior width ca. 0.2 mm. Color in ethanol pale yellow.

Prostomium globular and pear-shaped (Fig 7A), as long as wide. Eyes absent. One pair of clavate antennae inserted dorsolaterally, almost at prostomium base, 2/3 the length of prostomium (Fig 7A). Palps absent. Anterior chaetigers slightly wider and shorter than median and posterior ones.

Jaw apparatus not dissected. Mandibles butterfly-shaped medially fused, anterior region enlarged with smooth margins without free or fused teeth, posterior region slender and curved. Pair of maxillary carrier-like structures relatively large, basal plates with dentate inner margin; maxillary plates absent [17].

Two peristomial rings, both as long as prostomium, clearly well-defined dorsally and ventrally; posterior wider and longer than anterior and almost as long as the following chaetigers (Fig 7A).

Cylindrical parapodia, shorter in anterior, longer and narrower in posterior region. Dorsal cirrus absent. Papilliform ventral cirrus inserted medially in the parapodium, absent in the first one (Fig 7C).

Supra-acicular chaetae: (1) one long and serrated capillary, with a small limb anteriorly, longer and slender in posterior region; (2) one geniculate with short serrated margin (Fig 7C). Sub-acicular chaetae: (3) three compound heterogomph falcigers, distal end of shafts serrated and blades unidentate with serrated cutting edges: dorsalmost longest and ventralmost shortest; (4) one cultriform with serrated margin replacing the ventralmost compound in last nine chaetigers. Chaetae from median and posterior regions longer and slender (Fig 7C).

Pygidium rounded and narrower than previous chaetigers. Two pairs of clavate pygidial cirri, dorsal pair as long as pygidium, ventral pair half as long as pygidium (Fig 7B).

#### Remarks

Although the type material was in poor condition, the following differences from the original description were observed: presence of papilliform ventral cirrus inserted in the middle of the parapodium (Fig 7C), similar to *M*. *minuta*; absence of neuroacicula; presence of cultriform chaeta in some specimens, including the holotype.

Jumars [17] did not mention the presence of maxillary plates in the jaw apparatus, although more material is needed to better describe this structure.

*Meiodorvillea apalpata* resembles *Meiodorvillea* sp. B [19] in lacking palps, but the latter has dorsal cirri from chaetigers 2 to 4–5.

#### Geographic distribution and bathymetric range

Known only from the type locality, Northeastern Pacific Ocean, east coast of California (USA), 1,223–1,224 m, silty mud [17].

### *Meiodorvillea penhae* sp. nov.

**Fig 8:**
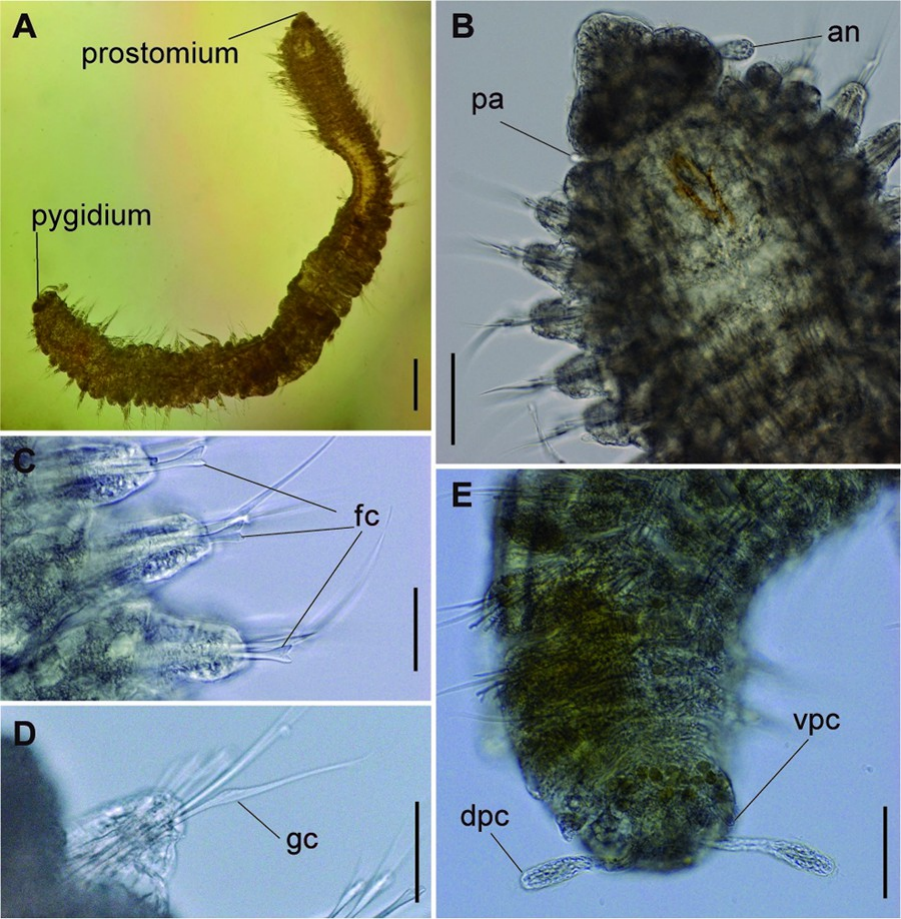
*Meiodorvillea penhae* sp. nov. (A) complete specimen, dorsal view; (B) anterior region, dorsal view; (C) parapodia from anterior region with furcate chaeta, dorsal view; (D) parapodium from posterior region with geniculate chaeta, dorsal view; (E) pygidial region, ventral view. Abbreviations: an = antenna; dpc = dorsal pygidial cirrus; fc = furcate chaeta; gc = geniculate chaeta; pa = palp; vpc = ventral pygidial cirrus. Scale bars: (A) = 200 μm; (B,D) = 100 μm; (C,E) = 50 μm.

**Fig 9:**
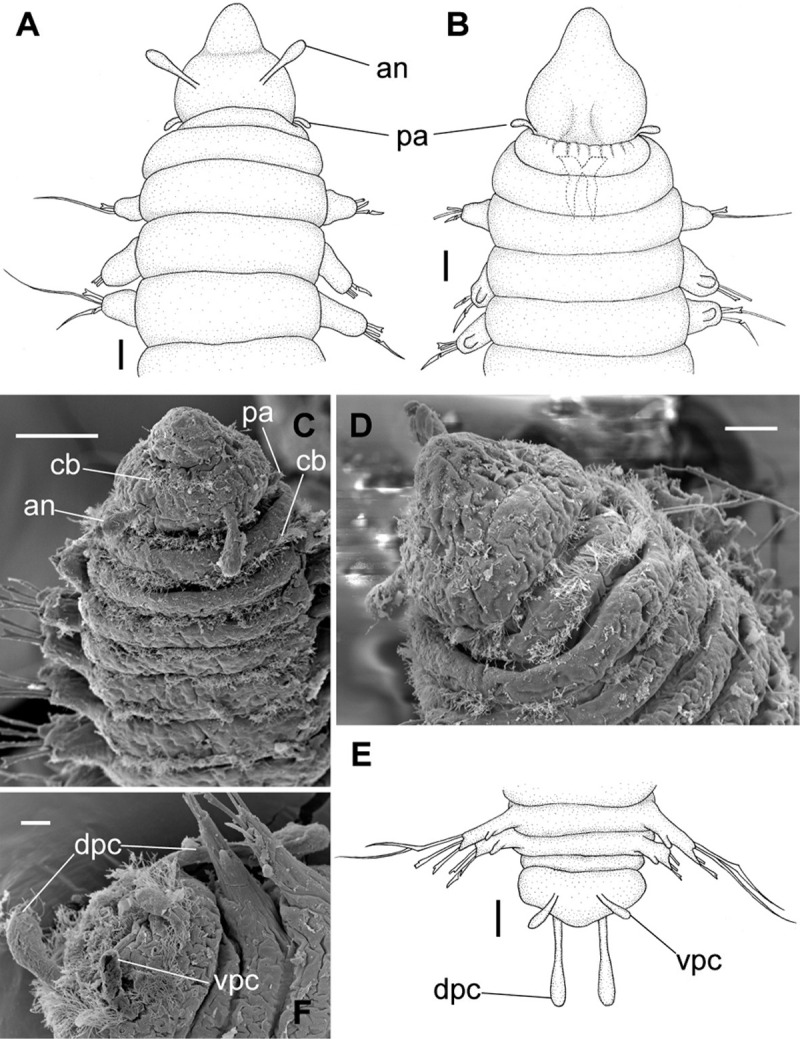
*Meiodorvillea penhae* sp. nov. (A,C) anterior region, dorsal view; (B) anterior region, ventral view; (D) anterior region, ventrolateral view; (E,F) pygidium, ventral view. Abbreviations: an = antenna; dpc = dorsal pygidial cirrus; pa = palp; vpc = ventral pygidial cirrus. Scale bars: (A,B,E) = 25 μm; (C) = 50 μm; (D) = 20 μm; (F) = 10 μm.

**Fig 10:**
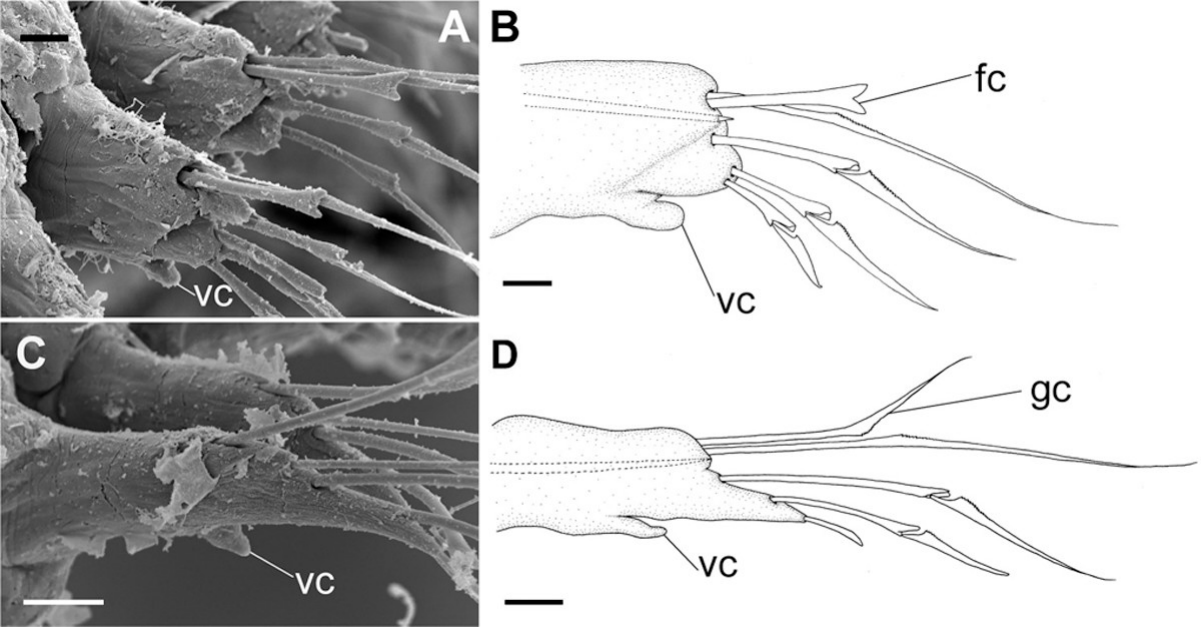
*Meiodorvillea penhae* sp. nov. (A,B) parapodia from anterior region; (C,D) parapodia from posterior region; all anterior view. Abbreviations: fc = furcate chaeta; gc = geniculate chaeta; vc = ventral cirrus. Scale bars: (A,C) = 15.6 μm; (B,D) = 10 μm.

**Fig 11:**
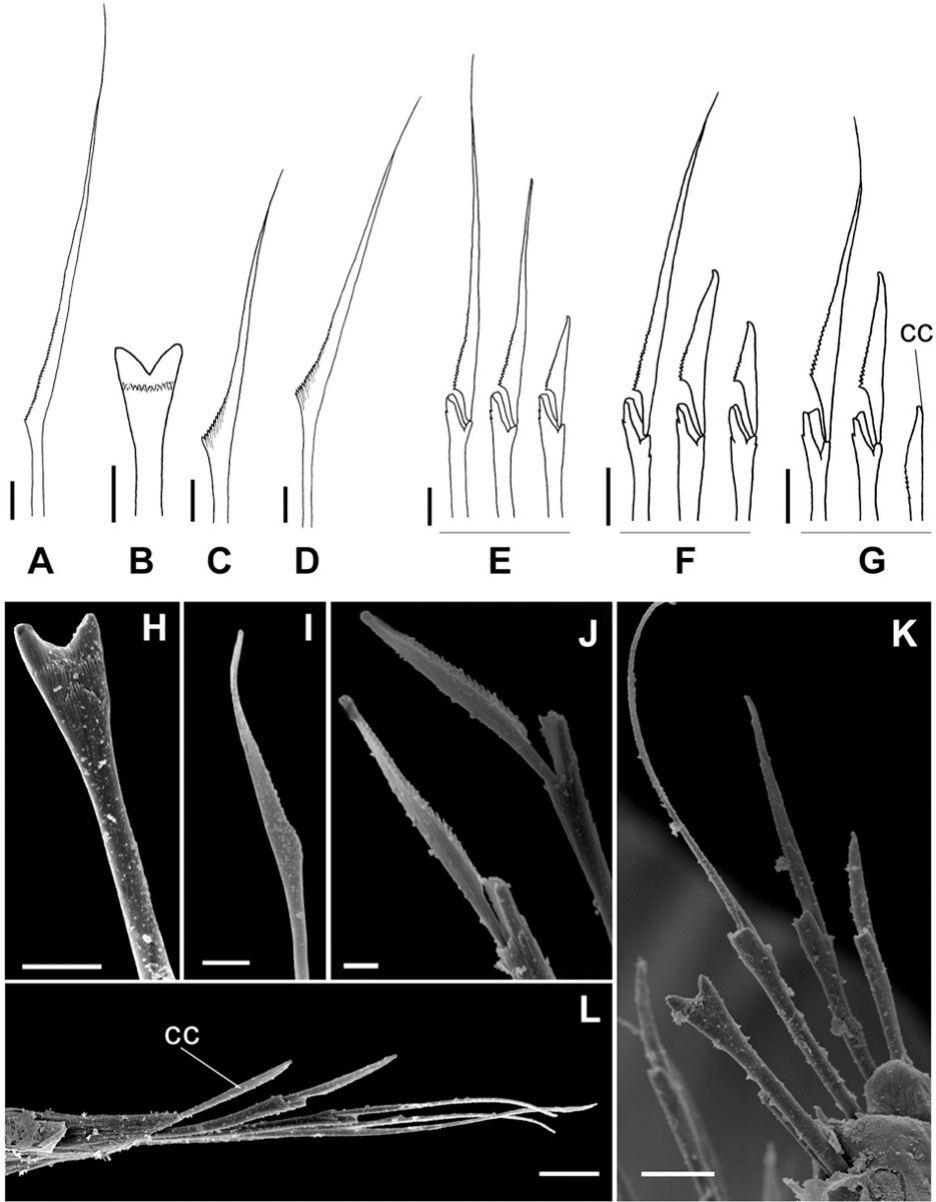
*Meiodorvillea penhae* sp. nov. (A) capillary chaeta, anterior region; (B,H) furcate chaeta; (C,D,I) geniculate chaeta;(E) compound chaetae, anterior region; (F) compound chaetae, median region; (G) compound and cultriform chaetae, posterior region; (J) compound chaetae; (K) chaetae, anterior region; (L) chaetae, posterior region. Abbreviations: cc = cultriform chaeta. Scale bars: (A-G) = 6.25 μm; (H-I) = 5 μm; (J) = 2 μm; (K-L) = 10 μm.

**Fig 12:**
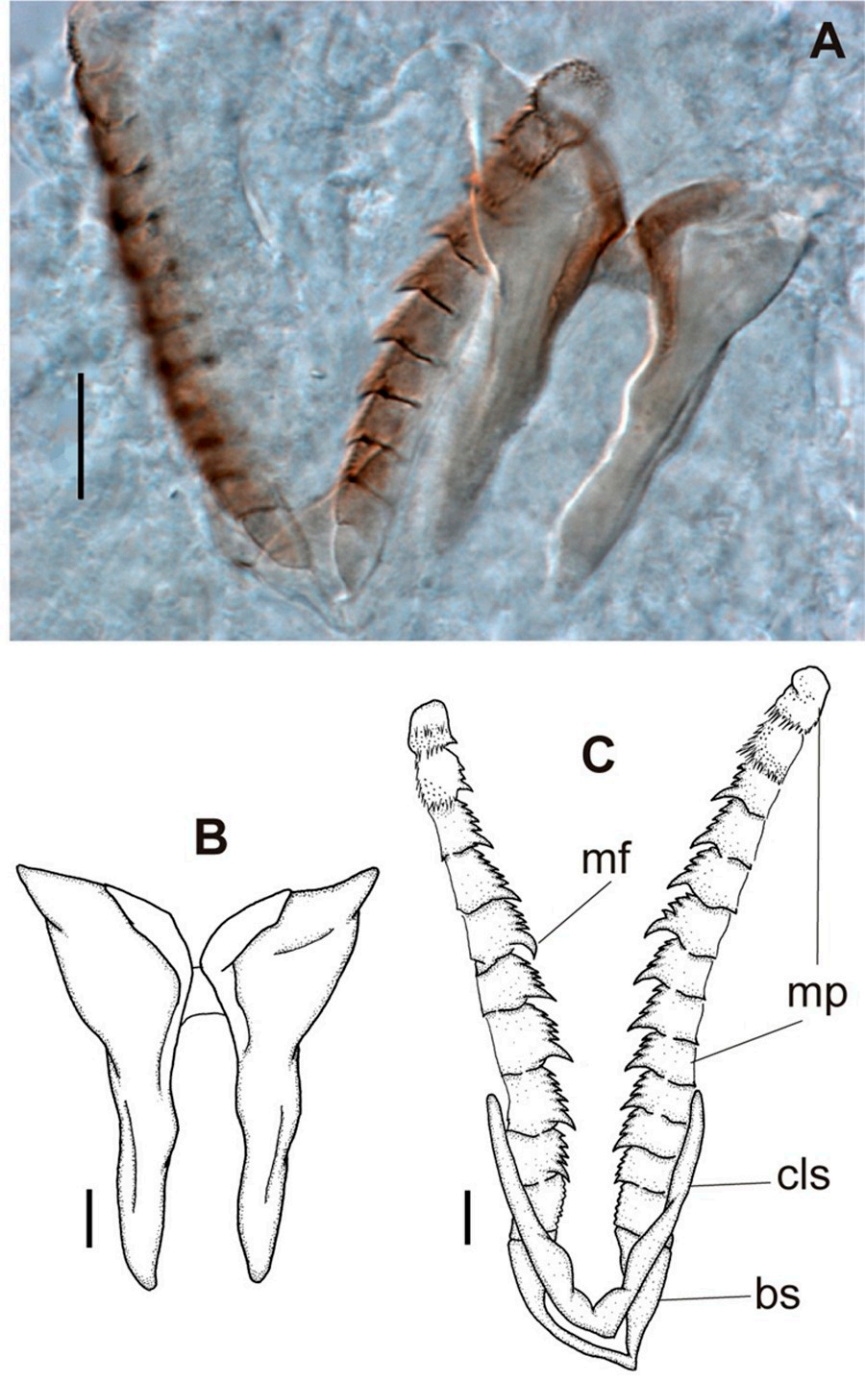
*Meiodorvillea penhae* sp. nov. (A) jaw apparatus; (B) mandibles; (C) maxillae; all dorsal view. Abbreviations: bs = basal plate; cls = carrier-like structure; mf = main fang; mp = maxillary plates. Scale bars: (A) = 20 μm; (B) = 10 μm; (C) = 6.25 μm.

(Figs 8–12, Table 1)

urn:lsid:zoobank.org:act:60CC2B5E-ED78-4497-85CD-BE63CFD14EF6

#### Type locality

Southwest Atlantic Ocean, 18°36’31.68"S 39°9’33"W, off Espírito Santo State, Brazil, 39 m depth.

#### Type material examined

Holotype: State of Espírito Santo–ZUEC-POL 21359 18°36’31.68"S 39°9’33"W, 39 m, sand, 17 Jan 2012. Paratypes: State of Espírito Santo–ZUEC-POL 21360 (3 specs/1 incomplete), 18°36’31.68"S 39°9’33"W, 39 m, sand, 17 Jan 2012; ZUEC-POL 21361 (4 specs) 20°12’21.46"S 39°58’0.3"W, 50 m, sand with biodetritus, 13 Jul 2013; ZUEC-POL 21362 (2 specs) 20°1’3.73"S 39°50’13.76"W, 53 m, sand with rhodoliths, 16 Dec 2010; ZUEC-POL 21363 (1 spec) 19°49’7.27"S 39°36’8.52"W, 158 m, muddy sand, 17 Jan 2012; ZUEC-POL 21364 (3 specs) 18°40’55.3"S 38°55’41.48"W, 55 m, sand, 17 Jan 2012. State of Rio de Janeiro–ZUEC-POL 21365 (5 specs) 21°22’58.61"S 40°19’41.01"W, 53 m, 21 Jul 2009. (m = meters depth).

#### Other material examined

State of Espírito Santo–ZUEC-POL 21366 (1 spec) 19°42’26.81"S 39°39’5.27"W, 39 m, sand, 15 Jul 2011; ZUEC-POL 21367 (1 spec) 19°41’24.99"S 39°31’20.42"W, 53 m, fine sand, 13 Jul 2011; ZUEC-POL 21368 (1 spec) 20°11’25.35"S 40°2’16.02"W, 39 m, mud, 20 Jan 2012; ZUEC-POL 21369 (1 spec) 19°37’41.83"S 39°35’31.52"W, 41 m, sand, 15 Jul 2011; ZUEC-POL 21370 (8 specs) 18°53’29.72"S 39°6’23.3"W, 52 m, sand, 18 Jan 2012; ZUEC-POL 21371 (10 specs/1 incomplete) 18°52’32.61"S 39°8’42.82"W, 40 m, muddy sand, 18 Jan 2012; ZUEC-POL 21372 (2 specs) 18°40’55.3"S 38°55’41.48"W, 55 m, sand, 17 Jan 2012; ZUEC-POL 21373 (1 spec) 20°34’53.42"S 40°6’27.43"W, 50 m, sand, 21 Jan 2012; ZUEC-POL 21374 (1 spec) 20°34’32.47"S 40°20’52.37"W, 25 m, mud, 21 Jan 2012; ZUEC-POL 21375 (2 specs) 19°26’5"S 39°17’38.92"W, 50 m, muddy sand, 19 Jan 2012; ZUEC-POL 21376 (3 specs) 21°4’1.29"S 40°18’50.11"W, 49 m, mud with biodetritus, 22 Jan 2012; ZUEC-POL 21379 (1 spec) 18°36’32.45"S 39°9’32.83"W, 40 m, sand, 02 Jul 2013; ZUEC-POL 21380 (1 spec) 18°40’57.41"S 38°55’39.92"W, 53 m, sand, 02 Jul 2013; ZUEC-POL 21381 (1 spec) 19°55’44.66"S 39°45’38.7"W, 51 m, rhodoliths with mud, 16 Dec 2010; ZUEC-POL 21382 (1 incomplete spec) 19°41’33.92"S 39°31’17.74"W, 54 m, sand, 12 Dec 2010; ZUEC-POL 21383 (1 spec) 21°10’16.281"S 40°45’58.437"W, 21 m, 22 Jul 2009; ZUEC-POL 21383 (2 specs) 19°9’51.36"S 39°29’20.53"W, 27 m, sand, 15 Jul 2013; ZUEC-POL 21385 (1 spec) 19°26’4.81"S 39°17’38.64"W, 50 m, muddy sand, 14 Jul 2013; ZUEC-POL 21386 (1 spec) 18°52’31.35"S 39°8’41.34"W, 40 m, calcareous, 15 Jul 2013; ZUEC-POL 21387 (1 spec) 20°34’34.37"S 40°20’50.77"W, 26 m, mud, 12 Jul 2013; ZUEC-POL 21388 (2 specs) 20°34’53.05"S 40°6’27.68"W, 49 m, sand with biodetritus, 12 Jul 2013; ZUEC-POL 21389 (1 spec) 19°52’35.48"S 39°49’5.63"W, 43 m, sand, 16 Dec 2010; ZUEC-POL 21390 (1 spec) 19°49’57.38"S 39°52’14.02"W, 33 m, muddy sand, 15 Dec 2010. State of Rio de Janeiro–ZUEC-POL 21377 (1 incomplete spec) 21°22’58.813"S 40°19’41.885"W, 52 m, 05 Mar 2009; ZUEC-POL 21378 (1 spec) 21°22’58.347"S 40°19’41.209"W, 53 m, 21 Jul 2009. (m = meters depth).

#### SEM material

ZUEC-POL 21391 (3 spec)–State of Espírito Santo, 18°36’31.68"S 39°9’33"W, 39 m, sand, 17 Jan 2012; State of Espírito Santo, 18°40’57.41"S 38°55’39.92"W, 53 m, sand, 02 Jul 2013; State of Rio de Janeiro, 21°22’58.975"S 40°19’42.346"W, 52 m, 05 Mar 2009. (m = meters depth).

#### Diagnosis

One pair of antennae and one pair of palps. Dorsal cirrus absent. Ventral cirrus papilliform, absent in the first chaetiger. Chaetae capillary, furcate thick and symmetrical on chaetigers 1 to 7–9, replaced by geniculate towards the end of body, dorsalmost compound spiniger or falciger, median and ventralmost falcigers, and cultriform in last chaetigers in some specimens, replacing the ventralmost compound. Two pairs of pygidial cirri.

#### Description of the holotype

Complete specimen, 45 chaetigers, 2.2 mm long, 0.21 mm wide in anterior region, 0.14 mm in posterior region, excluding parapodia. Width uniform, anterior region slightly wider (Fig 8A). Color in ethanol pale yellow.

Prostomium pear-shaped, as long as wide, anterior half depressed and rounded, posterior half globular, wider than the anterior half (Figs 8B and 9A–9D); ciliary band between them (Fig 9C). Eyes absent. One pair of clavate antennae inserted dorsolaterally on middle posterior half of prostomium, half as long as prostomium (Figs 8B, 9A and 9C). Pair of small and clavate palps inserted laterally at prostomium base, 1/2 the antennae length (Figs 8B and 9A–9C).

Two peristomial rings, both as long as prostomium when not retracted into prostomium (Figs 8B and 9A–9D); posterior wider than anterior, covering it dorsally. Transversal ciliary bands at base of peristomium and anterior chaetigers (Fig 9C).

Cylindrical parapodia, short and robust at anterior region (Figs 8C, 10A and 10B), tapering towards posterior region (Figs 8D, 10C and 10D), ciliary tufts dorsally on parapodia (Fig 10A). Dorsal cirrus absent. Papilliform ventral cirrus, inserted medially in the parapodium, larger in posterior region (Fig 10), absent in the first chaetiger.

Supra-acicular chaetae: (1) one long and serrated capillary (Fig 11A); (2) one thick furcate with short, stout, and symmetrical prongs with serrated base (Figs 8C, 11B, 11H and 11K), present in chaetigers 1 to 8, replaced by (3) one geniculate with serrated margin, shorter than capillary (Figs 8D, 11C, 11D and 11I). Sub-acicular chaetae: (4) three compound heterogomphs (Fig 11E–11G and 11J-11L), distal end of shafts serrated and blades unidentate with serrated cutting edge: dorsalmost longest with falcigerous or spinigerous blade, median facigerous, ventralmost falcigerous and shortest. Chaetae gradually slender and longer in posterior region. One internal thick acicula (Fig 10B and 10D).

Pygidium rounded and narrower than previous chaetigers. Two pairs of clavate pygidial cirri, dorsal pair twice as long as pygidium, ventral pair as long as pygidium (Figs 8E, 9E and 9F).

Jaw apparatus from additional non-type material with ventral and medially fused mandibles, anterior region enlarged with smooth margins without free or fused teeth, posterior region slender and curved (Fig 12A and 12B). Basal plates of maxillae with smooth inner margin, two subsymmetrical rows with 10 to 13 pairs of free rectangular and denticulate maxillary plates, each one with one posterior main fang and usually four anterior teeth, last and anteriormost plates larger and rounded, with small and more numerous teeth. (Fig 12A and 12C).

#### Variation

Complete specimens 1.67–3.09 mm long, 146–281 μm wide, 27–62 chaetigers. Anterior region of most specimens wider, some with moniliform segments on posterior region, some with both peristomial rings visible in dorsal view. Furcate chaeta from chaetigers 1 to 7–9. Many specimens, including paratypes, with cultriform chaeta randomly found on last chaetigers (Fig 11G and 11L), absent in holotype. Paired maxillary plates from 10 to 13 pairs.

#### Remarks

*Meiodorvillea penhae* **sp. nov.** has furcate chaeta in anterior region replaced by geniculate in median and posterior regions, differing from any other species of *Meiodorvillea*, but occurring in *Gymnodorvillea floridana* Wainright & Perkins, 1982 [31].

The poor condition of the specimens did not allow detecting the possible presence of basal ciliated bands in median and posterior chaetigers.

*Meiodorvillea penhae* **sp. nov.** resembles *M*. *minuta* in having antennae and palps of same length and as long as the prostomium. However, geniculate chaetae are only present in the former and dorsal cirri only in the latter. *Meiodorvillea apalpata* differs from both species in lacking palps and furcate chaetae. *Meiodorvillea penhae* **sp. nov.** apparently corresponds to morphotypes with rasper-like maxillary plates of *Meiodorvillea* sp. A, described by Wolf [19].

#### Geographic distribution and bathymetric range

Southwestern Atlantic Ocean, States of Espírito Santo and Rio de Janeiro (Brazil), 21 to 158 m, in sand, muddy sand, sand with biodetritus, sand with rhodoliths, mud, mud with biodetritus and rhodoliths with mud.

#### Etymology

The specific epithet “*penhae”* refers to Our Lady of Penha, patron saint of the State of Espírito Santo.

### *Meiodorvillea hartmanae* sp. nov.

**Fig 13:**
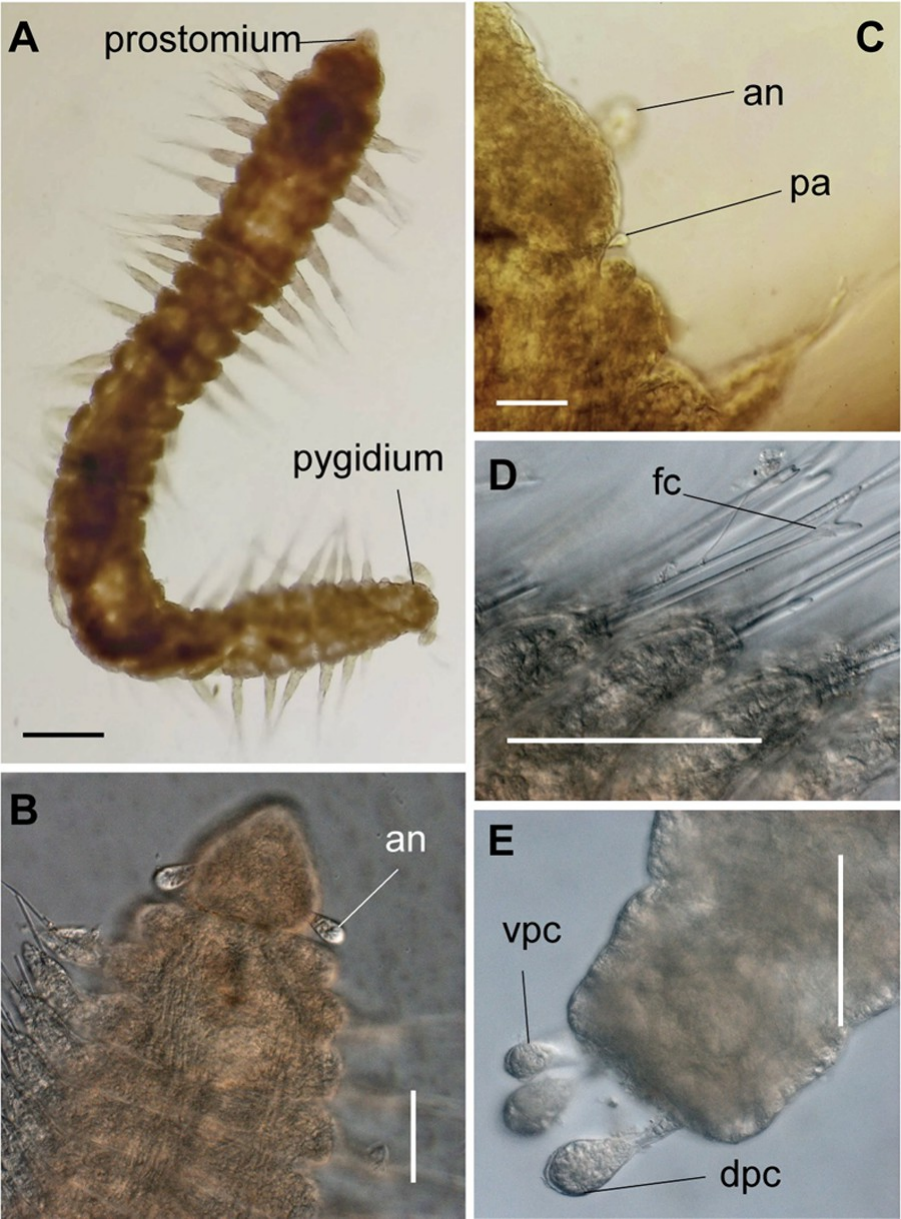
*Meiodorvillea hartmanae* sp. nov. (A) complete specimen, dorsal view; (B) anterior region, dorsal view; (C) anterior region, ventral view; (D) parapodia with furcate chaeta; (E) pygidium, dorsal view. Abbreviations: an = antenna; dpc = dorsal pygidial cirrus; fc = furcate chaeta; pa = palp; vpc = ventral pygidial cirrus. Scale bars: (A,B) = 100 μm; (C) = 31.25 μm; (D,E) = 88 μm.

**Fig 14:**
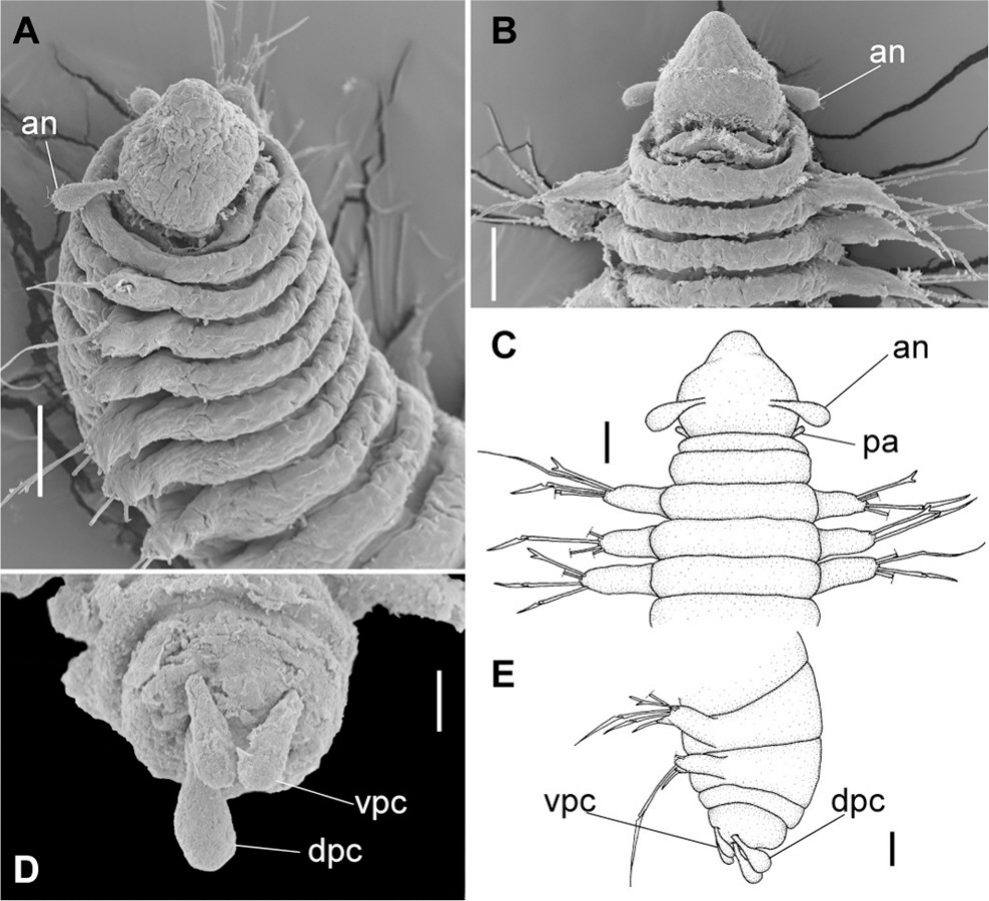
*Meiodorvillea hartmanae* sp. nov. (A) anterior region, ventrolateral view; (B) anterior region, ventral view; (C) anterior region, dorsal view; (D) pygidium, ventral view; (E) pygidium, lateral view. Abbreviations: an = antenna; dpc = dorsal pygidial cirrus; pa = palp; vpc = ventral pygidial cirrus. Scale bars: (A) = 40 μm; (B) = 25 μm; (C,E) = 31.25 μm; (D) = 20 μm.

**Fig 15:**
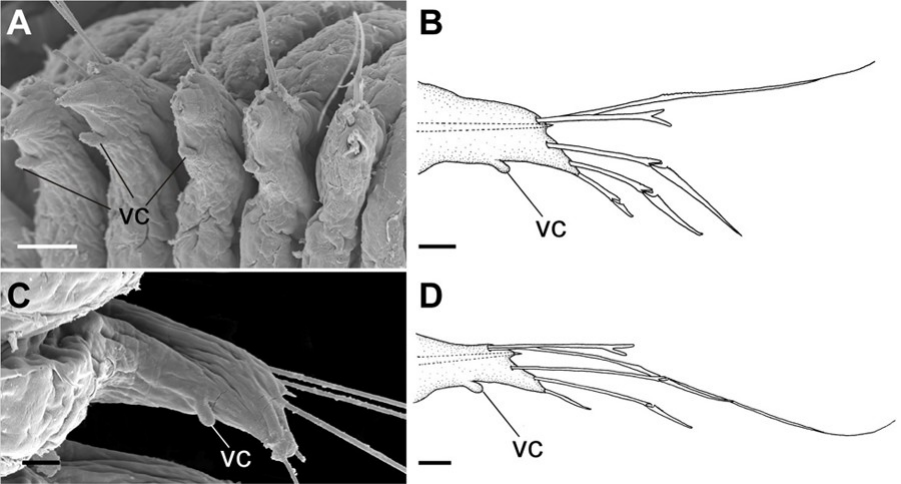
*Meiodorvillea hartmanae* sp. nov. (A,B) parapodia from anterior region, (A) ventrolateral view, (B) anterior view; (C,D) parapodia from posterior region, (C) ventral view, (D) anterior view. Abbreviations: vc = ventral cirrus. Scale bars: (A) = 20 μm; (B,D) = 15.6 μm; (C) = 10 μm.

**Fig 16:**
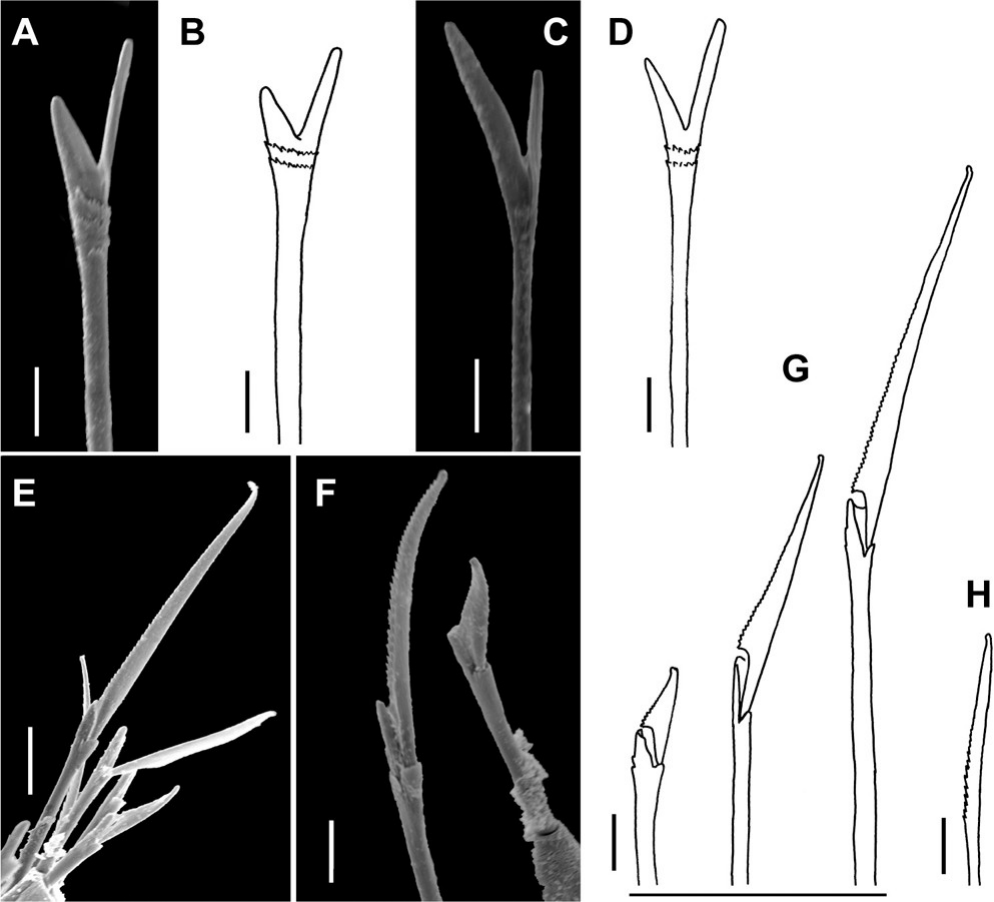
*Meiodorvillea hartmanae* sp. nov. (A,B) furcate chaeta, anterior region; (C,D) furcate chaeta, posterior region; (E,F,G) compound chaetae; (H) cultriform chaeta. Scale bars: (A,C,F) = 5 μm; (E) = 10 μm; (B,D,G,H) = 6.25 μm.

**Fig 17:**
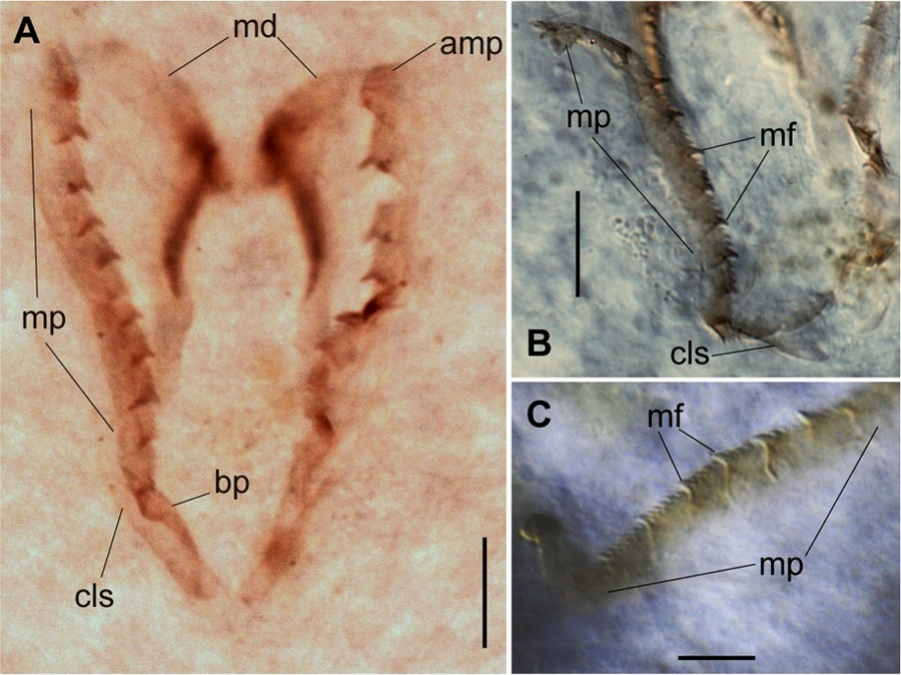
*Meiodorvillea hartmanae* sp. nov. (A) jaw apparatus; (B) maxillae; (C) maxillary plates; all dorsal view. Abbreviations: amp = anteriormost maxillary plates; bp = basal plate; cls = carrier-like structure; md = mandible; mf = main fang; mp = maxillary plates. Scale bars: (A,B) = 20 μm; (C) = 10 μm.

(Figs 13–17, Table 1)

urn:lsid:zoobank.org:act:9DC54BE0-27ED-44E2-AD4D-2CB22E05D830

#### Type locality

Southwest Atlantic Ocean, 23°15’11,480"S, 40°53’53,304"W, off Rio de Janeiro State, Brazil, 1,228.5 m depth.

#### Type material examined

Holotype: State of Rio de Janeiro–ZUEC-POL 21406 23°15’11.480"S 40°53’53.304"W, 1,228.5 m, 14 Jan 2009. Paratypes: State of Rio de Janeiro–ZUEC-POL 21407 (2 spec) 23°15’10.577"S 40°53’54.586"W, 1,300.7 m, 15 Jan 2009; ZUEC-POL 21408 (1 spec) 23°13’46.474"S 40°55’56.178"W, 986.4 m, 08 May 2008; ZUEC-POL 21409 (1 spec) 23°13’48.724"S 40°55’53.562"W, 1,001.4 m, 16 Jan 2009; ZUEC-POL 21410 (1 spec) 23°13’47.452"S 40°55’56.256"W, 986.4 m, 08 May 2008; ZUEC-POL 21411 (2 spec) 23°13’48.742"S 40°55’55.134"W, 986.4 m, 08 May 2008; ZUEC-POL 21412 (1 spec) 23°41’9.142"S 41°16’7.032"W, 1,001.5 m, 13 Jan 2009. (m = meters depth).

#### Other material examined

State of Rio de Janeiro–ZUEC-POL 21413 (1 spec) 21°45’54.702"S 39°59’27.418"W, 1,030 m, 06 Feb 2009; ZUEC-POL 21414 (1 spec) 23°13’48.868"S 40°55’54.397"W, 1,010.8 m, 16 Jan 2009; ZUEC-POL 21415 (1 spec) 23°15’11.480"S 40°53’53.304"W, 1,228.5 m, 14 Jan 2009; ZUEC-POL 21416 (1 spec) 21°55’7.018"S 39°54’31.298"W, 996.9 m, 07 Feb 2009. State of Espírito Santo–ZUEC-POL 21417 (1 spec) 21°4’37.64"S 40°8’32.68"W, 1,015 m, mud, 08 Jun 2013; ZUEC-POL 21418 (1 spec) 20°15’36.86"S 39°46’15.05"W, 1,040 m, sandy mud, 09 Jan 2012. (m = meters depth).

#### SEM material

State of Rio de Janeiro–ZUEC-POL 21419 (3 spec): 23°1’30.862"S 40°45’22.948"W, 964.8 m, 16 Jan 2009; 21°55’6.677"S 39°54’32.810"W, 997 m, 28 May 2008; 23°13’48.868"S 40°55’54.397"W, 1,010.8, 16 Jan 2009 (m = meters depth).

#### Diagnosis

One pair of antennae and one pair of palps. Dorsal cirri absent. Ventral cirrus papilliform, absent in the first chaetiger. Chaetae capillary, furcate asymmetrical, dorsalmost compound spiniger or falciger, median and ventralmost falcigers, and cultriform in last chaetigers in some specimens, replacing the ventralmost compound. Two pairs of pygidial cirri.

#### Description of the holotype

Complete specimen, 37 chaetigers, 1.9 mm long, anterior region 0.17 mm wide, excluding parapodia; body width uniform (Fig 13A), anterior region slightly wider. Color in ethanol pale yellow.

Prostomium pear-shaped, as long as wide, anterior half depressed and rounded, posterior half wider and globular (Figs 13A, 13B and 14A–14C). Eyes absent. One pair of clavate antennae inserted dorsolaterally on middle posterior half of prostomium, 1/3 of prostomium length (Figs 13B, 13C and 14A–14C). One pair of very small and clavate palps inserted laterally at prostomium base, difficult to see in dorsal view (Figs 13C and 14C).

Two peristomial rings, half as long as prostomium, posterior wider than anterior (Figs 13B and 14A-14C).

Cylindrical, long and stout parapodia, gradually tapering posteriorly (Fig 15). Dorsal cirrus absent. Small and papilliform ventral cirrus, inserted medially in the parapodium (Fig 15), absent in the first one.

Supra-acicular chaetae: (1) one long and serrated capillary; (2) one elongated and thin furcate, asymmetrical prongs in shape and size and serrated base (Fig 16A–16D). Sub-acicular chaetae: (3) three compound heterogomphs, dorsalmost longest with falcigerous or spinigerous blade, median falcigerous, ventralmost falcigerous and shortest; blades unidentate with serrated cutting edge (Fig 16E–16G); (4) one cultriform with serrated margin replacing the ventralmost compound in last chaetigers (Fig 16H). Chaetae gradually slender and longer along body. One internal thick acicula (Fig 15B and 15D).

Pygidium rounded and narrower than previous chaetigers. Two pairs of clavate pygidial cirri, dorsal pair twice as long as ventral pair (Figs 13E, 14D and 14E).

Jaw apparatus from additional non-type material with ventral and medially fused mandibles, anterior region enlarged with smooth margins without free or fused teeth, posterior region slender and curved (Fig 17A). Basal plates of maxillae with smooth inner margin, two subsymmetrical rows with 10–12 pairs of free rectangular and denticulate maxillary plates, each one with one posterior main fang and usually four anterior teeth last and anteriormost plates larger and rounded, with small and more numerous teeth. (Fig 17A–17C).

#### Variation

Complete specimens 1.5–2.9 mm long, maximum width 0.28 mm, 28–48 chaetigers; some with posterior moniliform segments (width equal to length). Palps visible only ventrally in some specimens. Most specimens with cultriform chaetae, with serrated cutting edge, in last 9–13 chaetigers, or randomly (Fig 16H). Maxillary plates varying from 10 to 13 pairs.

#### Remarks

*Meiodorvillea hartmanae* **sp. nov.** resembles *M*. *apalpata* and *M*. *penhae* in having papilliform ventral cirrus in all parapodia (except the 1^st^), but differs having furcate and lacking geniculate chaetae. On the other hand, *M*. *minuta* differs in having dorsal cirri on anterior chaetigers and a different shape of furcate.

*Meiodorvillea hartmanae* **sp. nov.** has smaller palps than all other species of the genus. In some specimens the palps are very difficult to see in dorsal view; in others, only ventrally. The species also has remnants of ciliary bands in the prostomium, peristomium, and chaetigers, as *M*. *penhae* and *M*. *minuta*. This character may possibly be shared by all species of *Meiodorvillea*.

#### Geographic distribution and bathymetric range

Southwestern Atlantic Ocean, States of Espírito Santo and Rio de Janeiro (Brazil), 964.8 to 1,300.7 m; mud, muddy sand, sandy mud.

#### Etymology

The specific epithet “*hartmanae*” honors Olga Hartman for her insight contributions to the taxonomy of Polychaeta and for describing the type species of *Meiodorvillea*.

### *Meiodorvillea jumarsi* sp. nov.

**Fig 18:**
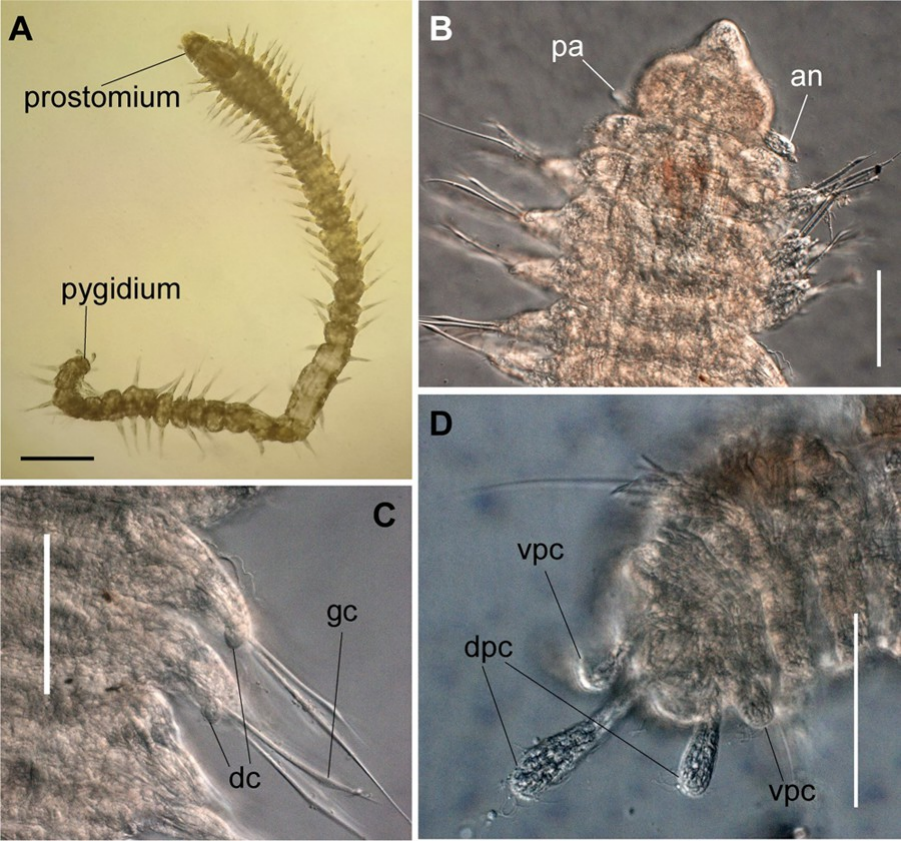
*Meiodorvillea jumarsi* sp. nov. (A) complete specimen, dorsal view; (B) anterior region, dorsal view; (C) parapodia with dorsal cirri and geniculate chaetae from anterior region, dorsal view; (D) pygidium, ventral view. Abbreviations: an = antenna; dc = dorsal cirrus; dpc = dorsal pygidial cirrus; gc = geniculate chaeta; md = mandible; mp = maxillary plates; pa = palp; vpc = ventral pygidial cirrus. Scale bars: (A) = 200 μm; (B) = 100 μm; (C,D) = 88 μm.

**Fig 19:**
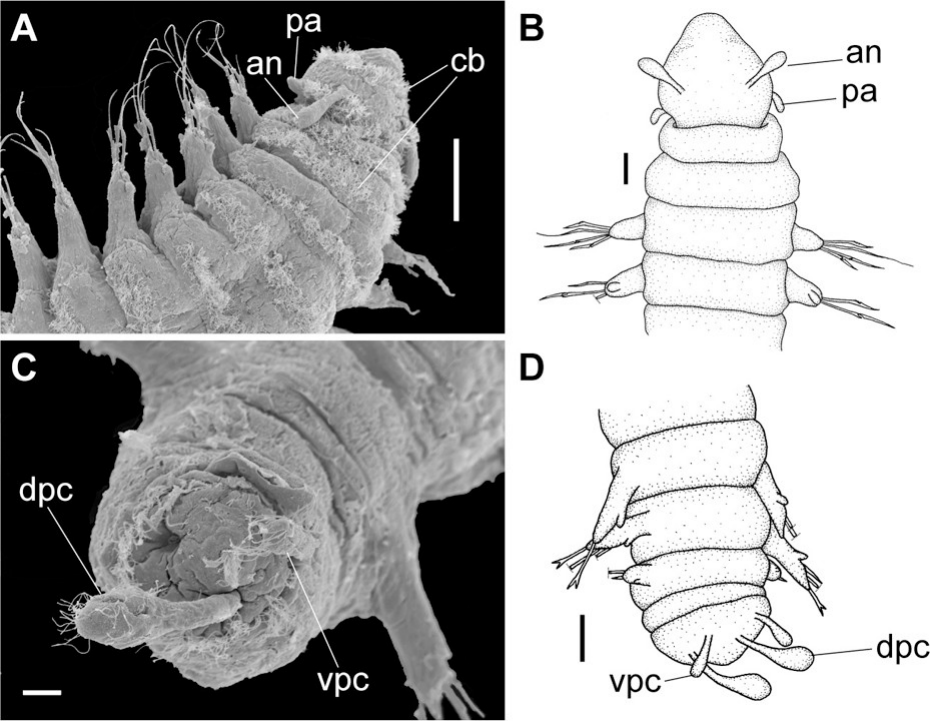
*Meiodorvillea jumarsi* sp. nov. (A) anterior region, dorsolateral view; (B) anterior region, dorsal view; (C,D) pygidium, ventral view. Abbreviations: an = antenna; dpc = dorsal pygidial cirrus; pa = palp; vpc = ventral pygidium cirrus. Scale bars: (A) = 50 μm; (B,D) = 25 μm; (C) = 10 μm.

**Fig 20:**
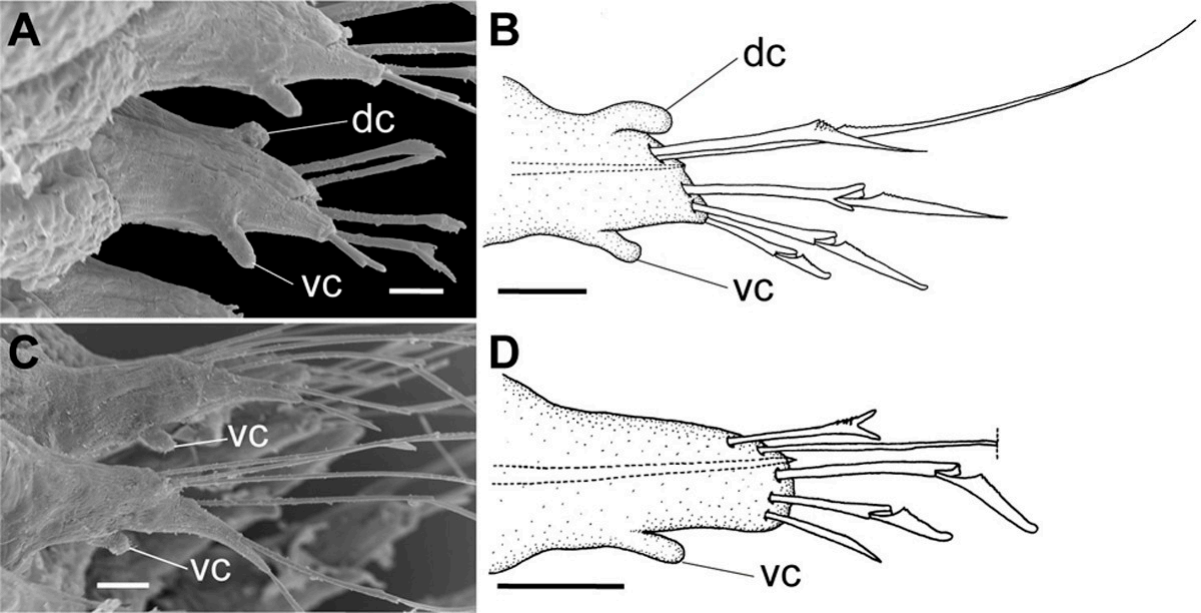
*Meiodorvillea jumarsi* sp. nov. (A,B) parapodia from anterior region; (C,D) parapodia from posterior region; all anterior view. Abbreviations: dc = dorsal cirrus; vc = ventral cirrus. Scale bars: (A,C) = 10 μm; (B,D) = 15.6 μm.

**Fig 21:**
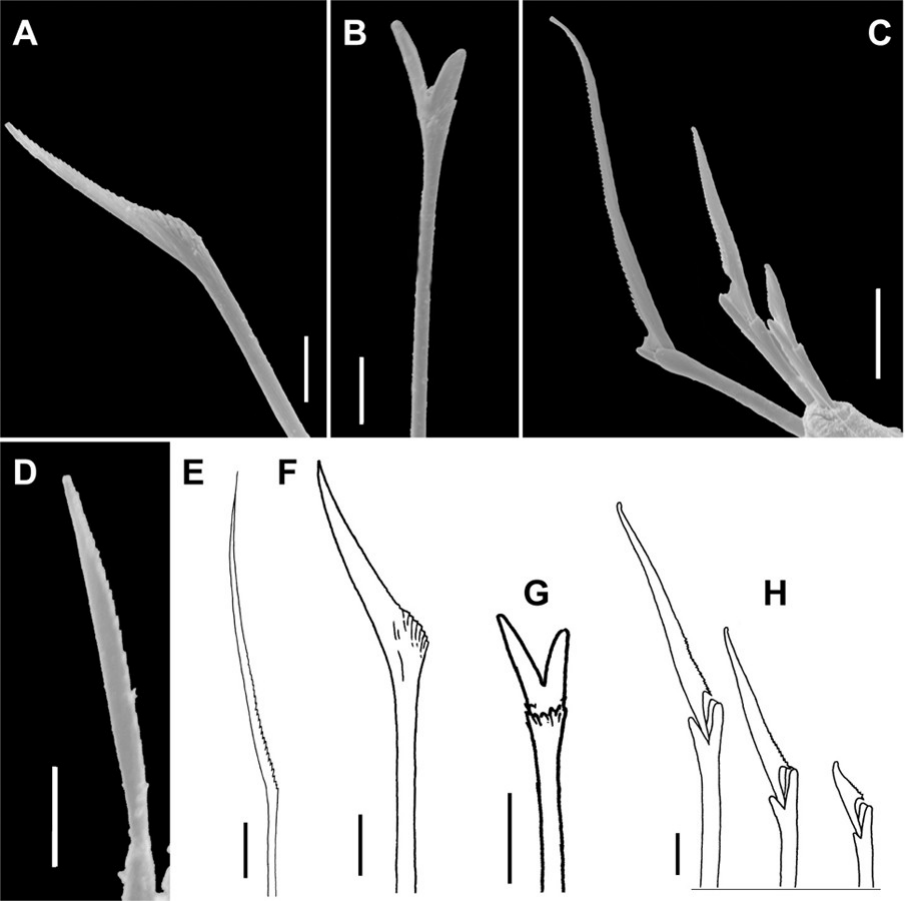
*Meiodorvillea jumarsi* sp. nov. (A,F) geniculate chaeta; (B,G) furcate chaeta; (C,H) compound chaetae; (D) cultriform chaeta; (E) capillary chaeta. Scale bars: (A,B,D) = 5 μm; (C) = 10 μm; (E) = 12.5 μm; (F,G,H) = 6.25 μm.

**Fig 22:**
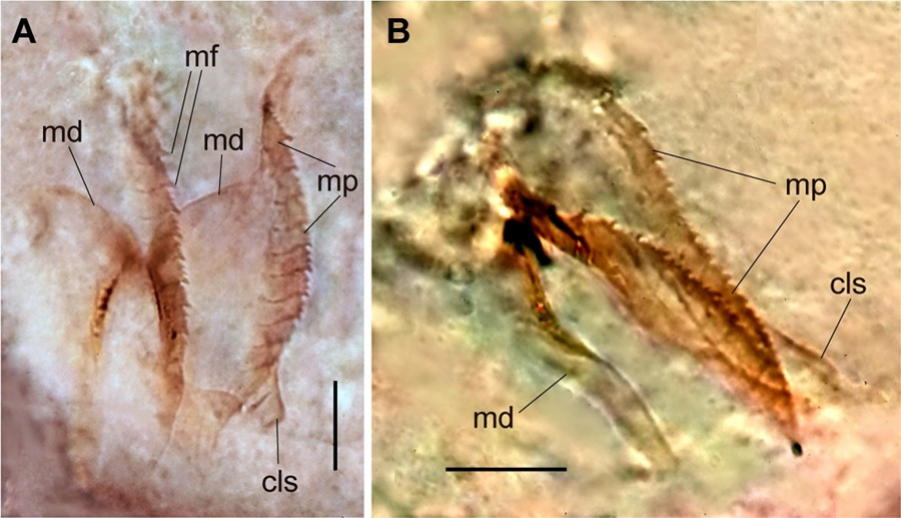
*Meiodorvillea jumarsi* sp. nov. (A,B) jaw apparatus, dorsal view. Abbreviations: md = mandibles; mf = main fang; mp = maxillary plates; cls = carrier-like-structure. Scale bars: (A,B) = 20 μm.

(Figs 18–22, Table 1)

urn:lsid:zoobank.org:act:E66DB9EC-5888-4CE7-B7E1-ACC24E06D779

#### Type locality

Southwest Atlantic Ocean, 19°41’29.14"S 39°31’18.18"W, off Espírito Santo State, Brazil, 53 m depth.

#### Type material examined

Holotype: State of Espírito Santo–ZUEC-POL 21420 19°41’29.14"S 39°31’18.18"W (ES), 53 m, sand, 14 Jul 2011. Paratypes: State of Espírito Santo: ZUEC-POL 21421 (1 spec) 19°41’33.92"S 39°31’17.74"W, 54 m, sand, 12 Dec 2010; ZUEC-POL 21422 (1 spec) 19°47’22.52"S 39°43’20.72"W, 41 m, sand, 15 Jul 2011; ZUEC-POL 21423 (1 spec) 19°52’29.66"S 39°49’8.1"W, 46 m, sand, 16 Jul 2011; ZUEC-POL 21424 (1 spec) 19°37’41.83"S 39°35’31.52"W, 41 m, sand, 15 Jul 2011; ZUEC-POL 21425 (1 spec) 19°41’29.14"S 39°31’18.18"W, 53 m, sand, 14 Jul 2011. (m = meters depth).

#### Other material examined

State of Espírito Santo–ZUEC-POL 21426 (1 spec) 20°1’2.6"S 39°50’18.72"W, 51 m, sand with rhodoliths, 17 Jul 2011. State of Rio de Janeiro–ZUEC-POL 21427 (1 spec) 22°59’0.677"S 40°48’28.837"W, 376.6 m, 31 Jan 2009; ZUEC-POL 21428 (2 spec) 22°59’1.044"S 40°48’27.955"W, 380.6 m, 31 Jan 2009; ZUEC-POL 21429 (1 spec) 23°10’23.820"S 40°56’45.497"W, 432 m, 01 Feb 2009. (m = meters depth).

#### SEM material

State of Espírito Santo–ZUEC-POL 21430 (3 spec): 19°41’29.14"S 39°31’18.18"W, 53 m, sand, 14 Jul 2011; 20°1’2.6"S 39°50’18.72"W, 51 m, sand with rhodoliths, 17 Jul 2011; 19°49’36.9"S 39°35’42.69"W, 378 m, mud, 28 Jun 2013 (m = meters depth).

#### Diagnosis

One pair of antennae and one pair of palps. Dorsal cirrus papilliform from chaetigers 2 to 5–9. Ventral cirrus papilliform, absent in the first chaetiger. Chaetae capillary, geniculate on chaetigers 1 to 7–13, replaced by furcate asymmetrical towards the end of body, dorsalmost compound spiniger or falciger and Y-shaped shafts in the first chaetigers, median and ventralmost falcigers, and cultriform in last chaetigers in some specimens, replacing the ventralmost compound. Two pairs of pygidial cirri.

#### Description of the holotype

Complete specimen, 39 chaetigers, 2.26 mm long, 0.84 mm wide in anterior region, excluding parapodia; body width uniform, anterior region slightly wider (Fig 18A). Color in ethanol pale yellow.

Prostomium pear-shaped, as long as wide, anterior half depressed and posterior half stout globular (Figs 18B, 19A and 19B); ciliary band between both (Fig 19A). Eyes absent. One pair of clavate antennae inserted dorsolaterally on middle posterior half of prostomium, 2/3 of prostomium length (Figs 18B, 19A and 19B). One pair of small and clavate palps inserted laterally at prostomium base, half as long as antennae (Figs 18B, 19A and 19B).

Two peristomial rings, both as long as the prostomium when not retracted, posterior wider and slightly longer than anterior dorsally (Figs 18B, 19A and 19B). Transversal ciliary bands at base of peristomium and anterior chaetigers (Fig 19A).

Cylindrical parapodia (Figs 18C and 20), gradually tapering posteriorly. Papilliform dorsal cirrus on chaetigers 2 to 5, inserted almost distally on parapodium (Figs 18C, 20A and 20B), absent thereafter. Papilliform ventral cirrus in all parapodia, absent in the first one, inserted medially in the parapodium (Fig 20C and 20D), shorter than dorsal when both present.

Supra-acicular chaetae: (1) one long and serrated capillary (Fig 21E); (2) one geniculate with serrated margin present in chaetigers 1 to 8 (Figs 18C, 21A and 21F), replaced by (3) one furcate with short and asymmetrical prongs in shape and size and serrated base (Fig 21B and 21G). Sub-acicular chaetae: (4) three compound heterogomphs (Fig 21C and 21H), dorsalmost longest with falcigerous or spinigerous blade and Y-shaped shaft (Fig 20B) in the first eight chaetigers, median falcigerous, ventralmost falcigerous and shortest; distal end of shafts serrated, blades unidentate and serrated cutting edge; (5) one cultriform with serrated margin replacing the ventralmost compound in last chaetigers (Fig 21D). All chaetae gradually slender and longer along body. One internal thick acicula (Fig 20B and 20D).

Pygidium rounded and narrower than previous chaetigers. Two pairs of clavate pygidial cirri, dorsal pair twice as long as pygidium, ventral pair 1.5 times as long as pygidium (Figs 18D, 19C and 19D).

Jaw apparatus from additional non-type material with ventral and medially fused mandibles, anterior region enlarged with smooth margins without free or fused teeth, posterior region slender and curved (Fig 22A). Basal plates of maxillae with smooth inner margin, two subsymmetrical rows with 10–12 pairs of free rectangular and denticulate maxillary plates, each one with one posterior main fang and usually four anterior teeth, last and anteriormost plates larger and rounded, with small and more numerous teeth. (Fig 22A and 22B).

#### Variation

Complete specimens 2.26–4.75 mm long, maximum width 0.22 mm, 36–59 chaetigers. Some specimens with posterior peristomial ring covering the anterior dorsally. Dorsal cirri from chaetiger 2 to 5–9 and geniculate chaeta in chaetigers 1 to 7–13.

#### Remarks

*Meiodorvillea jumarsi* **sp. nov.** differs from *M*. *apalpata* in having furcate and dorsal papilliform cirrus in anterior parapodia. The latter character also occurs in *M*. *minuta*, differing from the new species in lacking geniculate chaetae. *Meiodorvillea jumarsi* **sp. nov.** has geniculate chaetae in anterior parapodia replaced by furcate, while *M*. *penhae* has furcate replaced by geniculate chaetae in median and posterior regions. *Meiodorvillea jumarsi* **sp. nov.** also differs from *M*. *hartmanae* in having dorsal cirrus papilliform in anterior parapodia and from *Meiodorvillea* sp. B [19] in having palps.

#### Geographic distribution and bathymetric range

Southwestern Atlantic Ocean, States of Espírito Santo and Rio de Janeiro (Brazil), 41–432 m; mud, sand, sandy mud, muddy sand, sand with rhodoliths.

#### Etymology

The specific epithet “*jumarsi*” honors Peter A. Jumars for his insight contributions to the study of polychaetes and for first describing the genus *Meiodorvillea*.

A key to species of *Meiodorvillea* is presented below, as well as the Table 1 provides an overview of the main morphological features.

### Key to species of *Meiodorvillea* Jumars, 1974

1a- Palps absent. . . .. . . .. . . .. . . .. . . .. . . .. . . .. . . .. . . .. . . .. . . .. . . .. . . .. . . .. . . .. . . .. . . .. . . .. . . .. . . .. . .. . ..*Meiodorvillea apalpata*

1b- Palps present. . . .. . . .. . . .. . . .. . . .. . . .. . . .. . . .. . . .. . . .. . . .. . . .. . . .. . . .. . . .. . . .. . . .. . . .. . . .. . . .. . . .. . . .. . . .. . . .. . . .. . . .. . . .. . . .. . . .. . . ..2

2a- Dorsal cirri present. . . .. . . .. . . .. . . .. . . .. . . .. . . .. . . .. . . .. . . .. . . .. . . .. . . .. . . .. . . .. . . .. . . .. . . .. . . .. . . .. . . .. . . .. . . .. . . .. . . .. . . .. . . ..3

2b- Dorsal cirri absent. . . .. . . .. . . .. . . .. . . .. . . .. . . .. . . .. . . .. . . .. . . .. . . .. . . .. . . .. . . .. . . .. . . .. . . .. . . .. . . .. . . .. . . .. . . .. . . .. . . .. . . .. . .. . .4

3a- Supra-acicular geniculate chaetae absent. . . .. . . .. . . .. . . .. . . .. . . .. . . .. . . .. . . .. . .. . .*Meiodorvillea minuta*

3b- Supra-acicular geniculate chaetae in anterior chaetigers (1 to 7–13), then replaced by furcate. . . .. . . .. . . .. . . .. . . .. . . .. . . .. . . .. . . .. . . .. . . .. . . .. . . .. . . .. . . .. . . .. . . .. . . .. . .. . ..*Meiodorvillea jumarsi* **sp. nov.**

4a- Supra-acicular geniculate chaetae absent. . . .. . . .. . . .. . . .. . . .. . . ..*Meiodorvillea hartmanae* **sp. nov.**

4b- Supra-acicular furcate chaetae present in anterior chaetigers (1 to 7–9), then replaced by geniculate ……………………………………………..……. . .. . .*Meiodorvillea penhae* **sp. nov.**

## Discussion

This study provides the first record of *Meiodorvillea* from the Southwestern Atlantic Ocean, from a highly diverse and important protected area of the Brazilian coast. Our careful observations revealed new external morphological characters related to some appendages (presence and form of dorsal and ventral cirri and palps), chaetae (presence and form of furcate, geniculate and cultriform, as well as details of compound), and presence of ciliary bands, allowing to clarify the distinction between the previously known species, as well as to describe three new species. Conversely, new analyses of the jaw apparatus on a larger number of newly collected specimens are needed to understand this structure in each taxon, especially *M*. *apalpata*.

*Meiodorvillea* species show different bathymetric distributions (Table 1). *Meiodorvillea penhae* occurs between 21–158 meters depth, limited to continental shelf. *Meiodorvillea jumarsi* (41–432 meters depth), on the other hand, extends its distribution to slope areas, while *M*. *hartmanae* (964.8–1,300.7 m) and *M*. *apalpata* (1,223–1224 m) occur in deeper regions of the continental slope. The distribution of *M*. *minuta* (97–1050 meters depth) is more extensive, occurring from the continental shelf to deeper slope areas, both in the USA and in Brazil. However, the rare availability of collected material and few studies of this species are barriers to explain its disjoint distribution.

The taxonomy and phylogeny of Dorvilleidae remains unclear due to collecting difficulties, but also to the misclassification and omission of relevant morphological characters, especially from the jaw apparatus, a fundamental structure within Eunicida that, instead, did not show relevant differences among the known species of *Meiodorvillea*.

*Meiodorvillea* clusters with the phylogenetically closely related *Schistomeringos* Jumars, 1974 [17], *Dorvillea* Parfitt, 1866 [32], *Protodorvillea* Pettibone, 1961 [22], and *Eliberidens* Wolf, 1986 [17, 23, 31, 33], but also share certain morphological similarities with *Marycarmenia* Nuñez [24] and *Gymnodorvillea* Wainwright & Perkins, 1982 [31]. *Dorvillea*, *Schistomeringos*, and *Protodorvillea* differ from *Meiodorvillea* mainly due to length and shape of palps, presence of eyes, and quantity and morphology of chaetae. *Gymnodorvillea* differs from *Meiodorvillea* due to the absence of prostomial appendages and maxillary carriers, the last character shared with *Marycarmenia*, while *Eliberidens* has four rows of maxillae composed of superior and inferior basal plates and free maxillary plates absent. However, further phylogenetic studies are needed to clarify the inter-generic relationships.

## References

[1] Chamberlin RV. The Annelida Polychaeta. Memoirs of the Museum of Comparative Zoology at Harvard College. 1919; 48: 1–514. doi: 10.5962/bhl.title.49195

[2] Zanol J, Carrera-Parra LF, Steiner TM, Amaral ACZ, Wiklund H, Ravara A, et al. The Current State of Eunicida (Annelida) Systematics and Biodiversity. Diversity, 2021; 13(2): 74. doi: 10.3390/d13020074

[3] Claparède E, Mecznikow E. Beiträge zur Kenntnis der Entwickelungsgeschichte der Chaetopoden. Z Wiss Zool. 1869; 19: 163–205.

[4] Read G, Fauchald K. World Polychaeta database. Dorvilleidae Chamberlin, 1919. Accessed through: World Register of Marine Species. Available from: http://www.marinespecies.org/aphia.php?p=taxdetails&id=971.

[5] Tempestini A, Massamba-N’Siala G, Vermandele F, Beaudreau N, Mortz M, Dufresne F, et al. Extensive gene rearrangements in the mitogenomes of congeneric annelid species and insights on the evolutionary history of the genus *Ophryotrocha*. BMC Genomics. 2020; 21(1): 1–16. doi: 10.1186/s12864-020-07176-8 33225885 PMC7682095

[6] Ravara A, Wiklund H, Cunha MR. Four new species and further records of Dorvilleidae (Annelida, Polychaeta) from deep-sea organic substrata, NE Atlantic. Eur J Taxon. 2021; 736: 44–81. 10.5852/ejt.2021.736.1251.

[7] Moore JP. Polychaetous annelids from Monterey Bay and San Diego, California. Proc Acad Nat Sci Phila. 1909; 61: 235–295. 10.5852/ejt.2021.736.1251.

[8] Webster HE. The Annelida Chaetopoda of the Virginian coast. Transactions of the Albany Institute. 1879; 9: 202–269.

[9] Orensanz JM. Los anelidos poliquetos de la provincia biogeografica Argentina. III. Dorvilleidae. Physis Seccion A Los oceanos y sus organismos. 1973; 32(85): 325–342.

[10] Day JH. The polychaete fauna of South Africa. Part 8: New species and records from grab samples and dredging. Bulletin of the British Museum (Natural History), Series Zoology. 1963; 10(7): 381–445.

[11] McIntosh WC. On the structure of the British nemerteans, and some new British annelids. Transactions of the Royal Society of Edinburgh. 1869; 25(2): 305–433.

[12] Moore JP. Additional new species of Polychaeta from the North Pacific. Proc Acad Nat Sci Phila. 1906; 58: 217–260.

[13] Hartman O. Deep-water benthic polychaetous annelids off New England to Bermuda and other North Atlantic areas. Occasional Papers of the Allan Hancock Foundation. 1965; 28: 1–384.

[14] Ehlers E. Die Polychaeten des magellanischen und chilenischen Strandes. Ein faunistischer Versuch. Festschrift zur Feier des Hundertfünfzigjährigen Bestehens des Königlichen Gesellschaft der Wissenschaften zu Göttingen, Abhandlungen der Mathematisch-Physikalischen Klasse. 1901; 1–232, 25 plates.

[15] delle Chiaje S. Memorie sulla storia e notomia degli animali senza vertebre del Regno di Napoli. Stamperia della Societa’ Tipografica. Napoli. 1828; 3: 1–232.

[16] Amaral ACZ, Nallin SAH, Steiner TM, Forroni TO, Gomes Filho D. Catálogo das espécies de Annelida Polychaeta do Brasil. 2013. Available from https://www.ib.unicamp.br/museu_zoologia/sites/www.ib.unicamp.br.museu_zoologia/files/Catalogo_Polychaeta_Brasil_Amaral_et_al_2013_1a.pdf.

[17] Jumars PA. A generic revision of the Dorvilleidae (Polychaeta), with six new species from the deep North Pacific. Zool J Linn Soc. 1974; 54(2): 101–135. 10.1111/j.1096-3642.1974.tb00794.x.

[18] Hartmann-Schröder G. Die Polychaeten des Sublitorals. IN: Hartmann-Schröder G. and Gerd Hartmann, Zur Kenntnis des Sublitorals der chilenischen Küste unter besonderer Berücksichtigung der Polychaeten und Ostracoden. (Mit bemerkungen über den Einfluss sauerstoffarmer Strömungen auf die Besiedlung von marien Sedimenten.). Mitteilungen aus dem Hamburgischen zoologischen Museum und Institut. 1965; 62: 59–305.

[19] Wolf PS. Chapter 44. Dorvilleidae. In: Uebelacker JM, Johnson PG editors. Taxonomic Guide to the Polychaetes of the Northern Gulf of Mexico. Volume VI. B.A. Vittor & Associates, Inc. 1984; 44/1-44/37.

[20] Lavrado HP, Brasil AC. (Org.) Biodiversidade da região oceânica da Bacia de Campos: Macrofauna. 1. ed, Rio de Janeiro: SAG Serv. 2010; 232pp.

[21] Steiner TM, Santos CSG. A new species of *Neanthes* (Annelida, Polychaeta, Nereididae) from Brazil, and some remarks on *Neanthes bruaca* Lana & Sovierzoski, 1987. Beaufortia. 2004; 54(2): 39–57.

[22] Pettibone MH. New species of polychaete worms from the Atlantic Ocean, with a revision of the Dorvilleidae. Proc Biol Soc Wash. 1961; 74(19): 167–186.

[23] Eibye-Jacobsen D, Kristensen RM. A new genus and species of Dorvilleidae (Annelida, Polychaeta) from Bermuda, with a phylogenetic analysis of Dorvilleidae, Iphitimidae and Dinophilidae. Zool Scr. 1994; 23(2): 107–131.

[24] Núñez J. *Marycarmenia lysandrae*, a new genus and interstitial species (Polychaeta: Dorvilleidae) from Madeira. Bull Mar Sci. 1998; 62(1): 115–119.

[25] Oug E. New and lesser known Dorvilleidae (Annelida, Polychaeta) from Scandinavian and northeast American waters. Sarsia. 1978; 63(4): 285–303. 10.1080/00364827.1978.10411350.

[26] Wolf G. Morphologische Untersuchungen an den Kieferapparaten einiger rezenter und fossiler Eunicoidea (Polychaeta). Senckenbergische Naturforschenden Gesellschaft. 1980.

[27] Purschke G. Anatomy and ultrastructure of ventral pharyngeal organs and their phylogenetic importance in Polychaeta (Annelida). IV. The pharynx and jaws of the Dorvilleidae. Acta Zoologica. 1987; 68(2): 83–105. 10.1111/j.1463-6395.1987.tb00880.x.

[28] International Code of Zoological Nomenclature. Fourth Edition. The International Trust for Zoological Nomenclature, London. 1999.

[29] Blake JA. Vertical distribution of benthic infauna in continental slope sediments off Cape Lookout, North Carolina. Deep Sea Res 2 Top Stud Oceanogr. 1994; 41(4–6): 919–927. 10.1016/0967-0645(94)90054-X.

[30] Schaff TR, Levin LA. Spatial heterogeneity of benthos associated with biogenic structures on the North Carolina continental slope. Deep Sea Res 2 Top Stud Oceanogr. 1994; 41(4–6): 901–918. 10.1016/0967-0645(94)90053-1.

[31] Wainright SC, Perkins TH. *Gymnodorvillea floridana*, a new genus and species of Dorvilleidae (Polychaeta) from Southeastern Florida. Proc Biol Soc Wash. 1982; 95(4): 694–701.

[32] Parfitt E. Description of a *Nereis* new to science. Zoologist. 1866; 21: 113–114.

[33] Wolf PS. Four new genera of Dorvilleidae (Annelida: Polychaeta) from the Gulf of Mexico.Proc Biol Soc Wash. 1986; 99(4): 616–626.

